# Sexual and reproductive health and rights of migrant women attending primary care in England: A population-based cohort study of 1.2 million individuals of reproductive age (2009–2018)

**DOI:** 10.1016/j.jmh.2024.100214

**Published:** 2024-01-17

**Authors:** Neha Pathak, Claire X. Zhang, Yamina Boukari, Rachel Burns, Dee Menezes, Gregory Hugenholtz, Rebecca S French, Arturo Gonzalez-Izquierdo, Rohini Mathur, Spiros Denaxas, Andrew Hayward, Pam Sonnenberg, Robert W. Aldridge

**Affiliations:** a Institute of Health Informatics, University College London, London, NW1 2DA, UK; b Institute for Global Health, University College London, London, WC1E 6JB, UK; c Guy's & St Thomas's NHS Foundation Trust, London, SE1 9RT, UK; d National Perinatal Epidemiology Unit, Nuffield Department of Population Health, University of Oxford, OX3 7LF, UK; e Faculty of Public Health & Policy, London School of Hygiene & Tropical Medicine, London, UK; f Institute of Applied Health Research, University of Birmingham, Birmingham, B15 2TT, UK; g Wolfson Institute of Population Health, Queen Mary University of London, London, UK; h BHF Data Science Center, Health Data Research UK, London, NW1 2DA, UK; i Inclusion Health, UK Health Security Agency, London, UK; j Institute of Epidemiology and Healthcare, University College London, London, WC1E 7HB, UK; k Institute for Health Metrics and Evaluation, Seattle, WA, USA

**Keywords:** Migration health, Migrant, Migration, Sexual and reproductive health and rights, Sexual health, Reproductive health, SRHR, Electronic health records, Primary care

## Abstract

**Background:**

Evidence on the sexual and reproductive health and rights (SRHR) of migrants is lacking globally. We describe SRHR healthcare resource use and long-acting reversible contraceptives (LARCs) prescriptions for migrant versus non-migrant women attending primary care in England (2009–2018).

**Methods:**

This population-based observational cohort study, using Clinical Practice Research Datalink (CPRD) GOLD, included females living in England aged 15 to 49. Migration was defined using a validated codelist. Rates per 100 person years at risk (pyar) and adjusted rate ratios (RRs) were measured in migrants versus non-migrants for consultations related to all-causes, six exemplar SRHR outcomes, and LARC prescriptions. Proportions of migrants and non-migrants ever prescribed LARC were calculated.

**Findings:**

There were 25,112,116 consultations across 1,246,353 eligible individuals. 98,214 (7.9 %) individuals were migrants. All-cause consultation rates were lower in migrants versus non-migrants (509 vs 583/100pyar;RR 0.9;95 %CI 0.9–0.9), as were consultations rates for emergency contraception (RR 0.7;95 %CI 0.7–0.7) and cervical screening (RR 0.96;95 %CI 0.95–0.97). Higher rates of consultations were found in migrants for abortion (RR 1.2;95 %CI 1.1–1.2) and management of fertility problems (RR 1.39;95 %CI 1.08–1.79). No significant difference was observed for chlamydia testing and domestic violence. Of 1,205,258 individuals eligible for contraception, the proportion of non-migrants ever prescribed LARC (12.2 %;135,047/1,107,894) was almost double that of migrants (6.91 %;6,728/97,364). Higher copper intrauterine devices prescription rates were found in migrants (RR 1.53;95 %CI 1.45–1.61), whilst hormonal LARC rates were lower for migrants: levonorgestrel intrauterine device (RR 0.63;95 %CI 0.60–0.66), subdermal implant (RR 0.72;95 %CI 0.69–0.75), and progesterone-only injection (RR 0.35;95 %CI 0.34–0.36).

**Interpretation:**

Healthcare resource use differs between migrant and non-migrant women of reproductive age. Opportunities identified for tailored interventions include access to primary care, LARCs, emergency contraception and cervical screening. An inclusive approach to examining health needs is essential to actualise sexual and reproductive health as a human right.

## Research in context

### Evidence before this study

We searched the PubMed database using search terms based on the Guttmacher-*Lancet* commission definition of SRHR with no date restrictions: (migration OR migrant OR refugee OR asylum) AND (sexual health OR reproductive health OR reproductive cancers OR infertility OR gender-based violence OR HIV OR AIDS OR STIs OR abortion OR contraception OR maternal health OR newborn health) AND (England OR UK OR United Kingdom OR Britain). 40 studies were identified. Most published research on SRHR in migrants living in England was focused on HIV and STIs with a particular focus on migrants living with HIV. Only three studies used population-level data: two cohort studies of national HIV surveillance data reported that HIV in the UK was predominantly diagnosed in people born in Sub-Saharan Africa though this number is declining over time and one record linkage study reported that loss to HIV care follow up after giving birth was more common in Sub-Saharan African-born women compared to UK-born women. Studies of migrant women's SRHR focus mainly on maternal and newborn health. Only two small studies addressed contraceptive needs in migrants: one study of sexual health service use by Central and Eastern European migrants reported female migrants required family planning services more than non-migrants whilst a second qualitatively described cultural beliefs and concerns that shaped contraceptive choice in female asylum seekers. One only study addressed reproductive cancer, a small structured interview study which found that ethnic minority women who had migrated were more likely to be disengaged with cervical screening services. There were no studies of primary care and no studies using primary care national datasets.

### Added value of this study

Our study describes the rate of all-cause and SRHR consultations in migrant women living in England and registered with primary care, thereby addressing an important research gap in the literature. We provide estimates on a national scale with a large sample size allowing for adequate statistical power to look at less common outcomes with a high level of precision. For migrant women of reproductive age compared to non-migrants, consultation rates were lower for all-cause, emergency contraception and cervical screening whilst are higher for abortion and management of fertility problems indicating a difference in healthcare resource use between the groups. Migrant women of reproductive age are more likely to take up copper intrauterine devices in primary care compared to non-migrants and less likely to take up hormonal long-acting reversible contraception in primary care compared to non-migrants. These differences were impacted by certainty of migration status, ethnicity, and age/timing of cohort entry.

### Implications of all the available evidence

This study shows that migrants use primary care for sexual and reproductive healthcare differently when compared to non-migrants and may experience barriers to accessing services. This addresses an important gap in how national data is used to evaluate the delivery of SRHR in England so that it can guide expansion to meet the needs of vulernable and marginalised populations, a key recommendation of the Guttmacher-*Lancet* commission on SRHR. Tailored interventions in primary care that address specific barriers or promote specific facilitators for migrant groups are required to improve access to sexual and reproductive healthcare. To develop any such interventions, a co-production methodology is recommended to ensure that migrant and service providers' views are incorporated and to maximise impact.

## Introduction

Safe and fulfilling sex is a central part of the human experience and the Declaration of Sexual Rights states that everyone has a right to have sex free from the fear of disease, violence, or unplanned pregnancy (World Association of Sexual Health 2014). In 2018, the Guttmacher-*Lancet* Commission incorporated sexual rights in their definition of SHRH (Starrs et al., 2018) and highlighted that inadequate provision of sexual and reproductive healthcare is a violation of human rights. Their report identified international migrants as a large group with distinct SRHR needs that must be addressed to integrate SRHR into plans to achieve universal health coverage. The UCL-Lancet Commission on Migration and Health highlighted that addressing SRHR in migrants can be challenging due to the impact of abuse, exploitation and cultural norms across the migration trajectory on both SRHR access, experiences and outcomes (Abubakar et al., 2018). Where there is a clustering of needs such as gender and forced migration, individuals can be at greater risk of adverse SRHR outcomes including unwanted pregnancy and sexual violence (Kollodge, 2015).

In 2021, 1 in 6, or 10 million (16.8 %) residents in England and Wales were born outside of the UK. Existing studies of SRHR in migrants to the UK focus mainly on HIV/STIs. They describe multiple barriers to HIV testing and treatment by African migrants including language, stigma, and lack of service awareness (Shangase and Egbe, 2015; Fakoya et al., 2019). Studies of migrants from Central and Eastern Europe attending genitourinary clinics describe higher rates of high-risk sexual behaviours such as sex work, drug use, and condomless sex compared to non-migrants (Burns et al., 2009; Burns et al., 2011; Evans et al., 2011; Evans et al., 2011). One study interview explored barriers to cervical screening in women from ethnic minorities and found ethnic minority women who had migrated were more likely to be disengaged with services that UK-born ethnic minority (Marlow et al., 2015). However, these studies are limited by small sample sizes, sample representativeness, and do not provide national level data to guide policy and practice.

There are also no primary care studies of SRHR in migrants in the UK, despite primary care being an important part of the patient journey, particularly for women: cervical screening is largely completed in primary care (NHS Digital 2022), primary care is most commonly reported by women as the main source for obtaining contraceptive supplies in the UK (French et al., 2017), and national policies incentivise provision of long-acting reversible contraceptives (LARCs) in primary care (Ma et al., 2020). This means there is a need for large national scale studies of healthcare resource use in migrant women attending primary care that include a wide range of SRHR outcomes.

We aim to describe the rates of all-cause consultations, SRHR-related consultations, and long-acting reversible contraceptives (LARC) prescription rates in migrant women of reproductive age compared to non-migrant women registered with primary care in England. This will provide estimates of SRHR-specific healthcare resource use to identify gaps in SRHR provision for migrant women.

## Methods

### Study design

This is a population-based cohort study of linked Electronic Health Records (EHRs). The study is presented in accordance with the REporting of studies Conducted using Observational Routinely-collected Data (RECORD) statement reporting guidelines (Benchimol et al., 2015).

### Data sources

We used CPRD GOLD (Herrett et al., 2015; Denaxas et al., 2012) January 2019 build which comprised of 16,071,111 individuals in the UK (including transferred out and deceased individuals) that have been deemed acceptable by CPRD in terms of research quality. CPRD linked data sources included patient and practice postcode linked deprivation measures for the 2015 English Index of Multiple Deprivation (IMD). A detailed description with accompanying figure of data extraction, cleaning and linkage to create the final dataset for this study is provided in the Supplementary Appendix 1.

### **Primary outcomes, explanatory factors and covariates**

Our primary outcomes were all-cause and SHRH specific (abortion, emergency contraception, domestic violence and abuse, chlamydia testing, management of fertility problems, and cervical screening) primary care consultation rates, and LARC prescription rates. The explanatory factor was migration to the UK. This was defined using a migration clinical code list (Padmanabhan, 2017; Pathak, 2022a; Pathak et al., 2021). The following covariates were used in the analyses: age group during study year, deprivation status, primary care practice region, year of study, and ethnicity. Age groups and year were time-varying variables. We describe in detail how the explanatory factor and each covariate was derived in Supplementary Appendix 1. Our code lists used are freely available on GitHub (Pathak, 2022b).

### Study participant selection

The study population was based on participants fulfilling the following eligibility criteria: Recorded as female in CPRD GOLD; actively registered in CPRD GOLD between the ages of >= 15 or <=49 years old during the study period; the individual records are of ‘acceptable' research quality as verified by the CPRD; individual's primary care practice was deemed to be contributing ‘up-to-standard’ (UTS) data which refers to the overall quality of the data (Herrett et al., 2015). The study start date was 1st January 2009. The end of the study period was limited by the most recent data available in the January 2019 build of CPRD: 31st December 2018.

Participants entered the cohort at the earliest of: 1) 1st January 2009 if participant's start date of active data was before or on 1st January 2009, their active data ended after 1st January 2009, and the participant was aged >=15 and <=49 years old on 1st January 2009; 2) The start date of the participant's active data if this was after 1st January of 2009 and age at start date of participant's active data was >=15 and <= 49 years old; 3) the date of turning 15 years old if the date of turning 15 years old was before the end date of participant's active data and (a) start date of participant's active data was after 1st January 2009 and the participant was <15 years old on the start date of their active data OR (b) start of participant's active data was before or on the 1st January 2009 and the participant was < 15 years old on 1st January 2009.

Participants exited the cohort at the earliest of: 1) the end date of the participant's active data if this was before 31st December 2018 and participant was <50 years old on their end date of active data; 2) the date of turning 50 years old if the date the date of turning 50 years old was before the end date of participant's active data; 3) the last date of the study (31st December 2018).

#### Sub-cohorts for the LARC-specific analyses

Eligibility for using each of the LARCs differs based upon the UK Medical Eligibility Criteria (UK MEC) which we then applied to these sub-cohort analyses (Faculty of Sexual and Reproductive Health 2019).

To account for this, we created four open sub-cohorts for the LARC-specific analyses depending on the type of LARC prescribing being described: copper intrauterine device (Cu-IUD) cohort, levonorgestrel intrauterine device (LNG-IUD) cohort, subdermal Implant (SDI) cohort, progestogen-only injectable (POI) cohort.

For this study, participants were not eligible if they had a condition falling into UK MEC 3 or 4, or if a participant had undergone hysterectomy or sterilisation. Some conditions (e.g. pregnancy, sexually transmitted infections) are temporary meaning that a participant could be censored from the cohort during that time. Table 1 summarises the UK MEC 3 & 4 conditions for each type of LARC and then subsequent eligibility criteria applied in this study.

**Table 1:** LARC-specific eligibility criteria for study of LARC prescription rates in migrant women versus non-migrant women (CPRD GOLD, 2009–2018).

LARC Type	UK MEC 3 & 4 conditions	Conditions used for study eligibility	Conditions not used for study eligibility
Cu-IUD	Postnatal < 4 weeks; cervical cancer; endometrial cancer; malignant gestational trophoblastic disease; current pelvic inflammatory disease; current chlamydia; current gonorrhoea; pelvic tuberculosis; complicated organ transplant; HIV with CD4 <200; uterine distortion; known long QT; unexplained vaginal bleeding.	Postnatal < 4 weeks; cervical cancer; endometrial cancer; malignant gestational trophoblastic disease; current pelvic inflammatory disease; current chlamydia; current gonorrhoea; pelvic tuberculosis.	Complicated organ transplant, HIV with CD4 <200, uterine distortion, known long QT, unexplained vaginal bleeding - due to the lack of a suitable primary care codelist that exists or could be developed.
LNG-IUD	Postnatal < 4 weeks; cervical cancer; endometrial cancer; malignant gestational trophoblastic disease; current pelvic inflammatory disease; current chlamydia; current gonorrhoea; pelvic tuberculosis; severe decompensated cirrhosis; hepatocellular adenoma; malignant hepatocellular adenoma; breast cancer; complicated organ transplant; HIV with CD4 <200; uterine distortion; known long QT; unexplained vaginal bleeding.	Postnatal < 4 weeks; cervical cancer; endometrial cancer; malignant gestational trophoblastic disease; current pelvic inflammatory disease; current chlamydia; current gonorrhoea; pelvic tuberculosis; severe decompensated cirrhosis; hepatocellular adenoma; malignant hepatocellular adenoma; breast cancer.	Complicated organ transplant, HIV with CD4 <200, uterine distortion, known long QT, unexplained vaginal bleeding - due to the lack of a suitable primary care codelist that exists or could be developed.
SDI	Severe decompensated cirrhosis; hepatocellular adenoma; malignant hepatocellular adenoma; breast cancer; unexplained vaginal bleeding; multiple cardiovascular risk factors.	Severe decompensated cirrhosis; hepatocellular adenoma; malignant hepatocellular adenoma; breast cancer.	Unexplained vaginal bleeding; multiple cardiovascular risk factors - due to the lack of a suitable primary care codelist that exists or could be developed.
POI	Vascular disease; ischaemic heart disease; stroke (cerebrovascular accident and/or transient ischaemic attack); malignant hepatocellular adenoma; breast cancer; unexplained vaginal bleeding; multiple cardiovascular risk factors.	Vascular disease; ischaemic heart disease; stroke (cerebrovascular accident and/or transient ischaemic attack); malignant hepatocellular adenoma; breast cancer.	Unexplained vaginal bleeding; multiple cardiovascular risk factors - due to the lack of a suitable primary care codelist that exists or could be developed.

Cu-IUD = Copper intrauterine device; LNG-IUD = levonorgestrel intrauterine devices; SDI = subdermal implant; POI = progestogen-only injection.

### Analysis

We describe the number of individuals, percentage of the population and person years were measured and reported for migrants and non-migrants for the following baseline characteristics: age group at cohort entry, practice region, IMD, ethnicity and year of the study. A description of temporal trends was also completed. For all outcomes, we describe the number of events, mean number of events per individual plus standard deviation (SD), median number of events per individual plus interquartile range (IQR). For the LARC sub-cohorts, we also describe the proportion of non-migrants who had ever been prescribed any type of LARC and the most frequently prescribed LARC.

Consultation rates and LARC Prescription rates were calculated for all outcomes in migrants and non-migrants stratified by year, age category during the study year (not at cohort entry), ethnicity, practice region, and IMD. Rate ratios were generated using negative binomial regression with non-migrants as the reference group. Our multivariable model adjusted for year, age category, IMD, and primary care practice region.

All data analysis was completed in using R studio with all code freely available on GitHub (Ma et al., 2020).

### Sensitivity analyses and missing data

We undertook several sensitivity analyses in order to investigate potential sources of bias including stratification by certainty of migration status to address misclassification bias; stratification by ethnicity to address the lack of representativeness of the migration codelist for ethnic groups; a matched cohort analysis with 12-month washout to examine bias from secular trends present in the way migrants enter the cohort; assuming minimum pregnancy durations to address missing data on pregnancy duration. We undertook a complete case analysis, describing completeness for ethnicity and IMD variables in the migrant cohort and the non-migrant cohort.

### Ethics

A research protocol was developed and approved by the Medicines and Healthcare products Regulatory Agency (MHRA) UK Independent Scientific Advisory Committee (ISAC protocol 19_062R). This study was carried out as part of the CALIBER programme (Denaxas et al., 2012) (Denaxas et al., 2019) and set out in an open access protocol (Pathak et al., 2020). CALIBER has research ethics approval (09/H0810/16) and ECC approval (ECC 2-06(b)/2009 CALIBER dataset) and initial funding was provided by the Wellcome Trust (086091/Z/08/Z) and the National Institute for Health Research (RP-PG-0407- 10,314).The funders had no role in study design, data collection and analysis, decision to publish, or preparation of the manuscript.

## Results

**Fig. 1:**
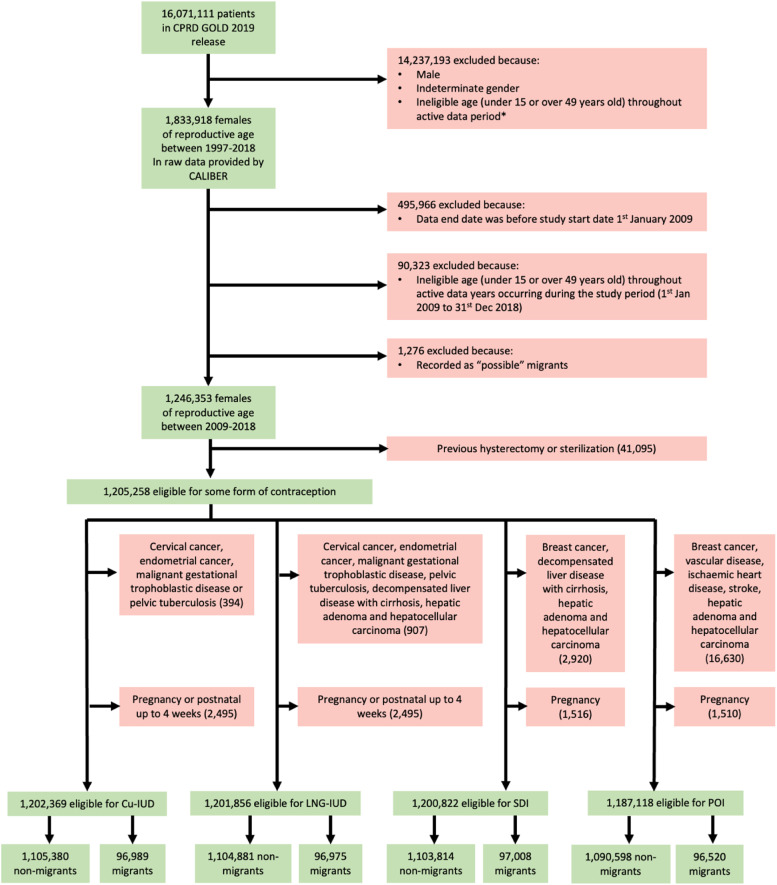
Flow diagram of study population selection to create eligible cohorts (2009–2019).

1246,353 out of 16,071,111 (7.8 %) individuals in the January 2019 release of CPRD GOLD had active data with a general practice in England between 1st January 2009 and 31st December 2018 and were aged 15 to 49 (Fig. 1). 3.3 % (41,095/1246,253) were excluded for the LARC sub-cohort due to evidence of previous hysterectomy or sterilisation. 96.7 % (1205,258/1246,353) were eligible for any form of contraception between 1st January 2009 and 31st December 2018. From this any-contraception cohort, 99.8 % (1202,369/1205,258) were eligible for Cu-IUDs, 99.7 % (1201,856/1205,258) were eligible for LNG-IUDs, 99.6 % (1200,822/1205,258) were eligible for SDIs, and 98.5 % (1187,118/1205,258) were eligible for POIs.

CPRD = Clinical Practice Research Datalink; Cu-IUD = copper intrauterine device; LNG-IUD = levonorgestrel intrauterine devices; SDI = subdermal implant; POI = progestogen-only injectable contraceptives

The distribution of baseline characteristics of all study participants and person years at risk (pyar) contributing to the study are outlined in Table 2. For non-migrants, age group at cohort entry was highest for the 15- to 19-year-old age group (210,026/1148,139; 18.3 %) followed by 25–29 year olds (187,619/1148,139; 16.3 %). For migrants, age group at cohort entry was highest for 25–29 year olds (25,139/98,214; 25.6 %) followed by 30–34 year olds (20,894/98,214; 21.3 %). Ethnicity was most commonly classed as unknown for non-migrants (423,398/1148,139; 36.9 %) followed by White British (267,431/1148,139; 23.3 %). For migrants, ethnicity was most commonly classed as White Non-British (41,718/98,214;42.5 %) followed by Asian/Asian British (24,939/98,214; 25.4 %). The proportion of individuals with unknown ethnicity was lower for migrants (10,976/98,214; 11.2 %) compared to non-migrants (423,398/1148,139; 36.9 %).

**Table 2:** Baseline demographic characteristics of study participants stratified by migration status (2009–2018).

		CPRD⁎⁎			
Baseline characteristic		Non-migrants		Migrants	
		No. Individuals ( %)	Pyar⁎⁎⁎	No. Individuals ( %)	Pyar
Totals*		1148,139 (92.1 %)	4072,942	98,214 (7.9 %)	270,326
Age group at cohort entry	15–19 years	210,026 (18.3 %)	706,451	8601 (8.8 %)	23,956
	20–24 years	173,257 (15.1 %)	537,864	15,598 (15.9 %)	37,115
	25–29 years	187,619 (16.3 %)	616,461	25,139 (25.6 %)	65,372
	30–34 years	167,895 (14.6 %)	627,229	20,894 (21.3 %)	59,326
	35–39 years	151,578 (13.2 %)	665,527	13,820 (14.1 %)	43,826
	40–44 years	140,015 (12.2 %)	625,796	8858 (9.0 %)	29,328
	45–49 years	117,749 (10.3 %)	293,614	5304 (5.4 %)	11,403
Ethnicity	White British	267,431 (23.3 %)	986,246	1399 (1.4 %)	3718
	White Non-British	113,468 (9.9 %)	361,192	41,718 (42.5 %)	112,251
	Mixed	13,816 (1.2 %)	42,608	2600 (2.6 %)	6904
	Asian/Asian British	51,192 (4.5 %)	168,868	24,939 (25.4 %)	78,002
	Black/Black British	35,073 (3.1 %)	117,136	9080 (9.2 %)	28,291
	Other	243,064 (21.2 %)	920,495	7020 (7.1 %)	21,134
	Unknown	423,398 (36.9 %)	1476,396	10,976 (11.2 %)	20,025
Practice region	North East	17,832 (1.6 %)	70,393	1221 (1.2 %	2972
	North West	148,286 (12.9 %)	576,482	8253 (8.4 %)	25,758
	Yorkshire & The Humber	29,508 (2.6 %)	91,678	484 (0.5 %)	1263
	East Midlands	27,102 (2.4 %)	56,119	528 (0.5 %)	676
	West Midlands	119,424 (10.4 %)	445,086	5995 (6.1 %)	17,729
	East of England	124,684 (10.9 %)	413,388	7186 (7.3 %)	19,767
	South West	138,515 (12.1 %)	468,924	6271 (6.4 %)	18,193
	South Central	159,554 (13.9 %)	581,855	12,384 (12.6 %)	25,923
	London	219,501 (19.1 %)	697,727	46,753 (47.6 %)	129,530
	South East Coast	163,733 (14.3 %)	671,289	9139 (9.3 %)	25,758
Index of Multiple Deprivation (IMD)	1 (Least deprived)	256,908 (22.4 %)	960,536	11,332 (11.5 %)	28,773
	2	240,701 (21.0 %)	846,926	14,099 (14.4 %)	37,644
	3	234,940 (20.5 %)	814,457	18,123 (18.5 %)	46,553
	4	222,027 (19.3 %)	772,385	26,887 (27.4 %)	74,480
	5 (Most deprived)	193,563 (16.9 %)	678,638	27,773 (28.3 %)	82,876
Year of cohort entry	2009	630,742 (54.9 %)	2810,470	27,840 (28.3 %)	114,385
	2010	83,479 (7.3 %)	290,116	8997 (9.2 %)	30,391
	2011	84,114 (7.3 %)	265,716	9686 (9.9 %)	29,312
	2012	82,630 (7.2 %)	221,334	11,202 (11.4 %)	27,289
	2013	77,393 (6.7 %)	173,289	9562 (9.7 %)	22,879
	2014	65,617 (5.7 %)	131,143	8246 (8.4 %)	17,079
	2015	47,559 (4.1 %)	83,977	9709 (9.9 %)	13,523
	2016	33,210 (2.9 %)	56,056	5259 (5.4 %)	8985
	2017	24,046 (2.1 %)	30,988	4028 (4.1 %)	4907
	2018	19,349 (1.7 % %)	9851	3685 (3.8 %)	1576
Year of study	2009	630,742 (54.9 %)	565,850	27,840 (28.3 %)	22,248
	2010	637,949 (55.6 %)	561,293	33,417 (34 %)	26,848
	2011	622,909 (54.3 %)	546,423	37,289 (38 %)	30,188
	2012	607,940 (53 %)	541,821	42,931 (43.7 %)	34,990
	2013	590,864 (51.5 %)	505,626	45,872 (46.7 %)	36,794
	2014	522,384 (45.5 %)	432,781	41,168 (41.9 %)	31,879
	2015	419,487 (36.5 %)	341,218	38,533 (39.2 %)	28,108
	2016	297,474 (25.9 %)	241,865	28,572 (29.1 %)	22,764
	2017	228,998 (19.9 %)	190,046	23,810 (24.2 %)	19,796
	2018	182,333 (15.9 %)	12,019	22,694 (23.1 %)	16,710

⁎ column percentages for all variables except for the first row which is a row percentage and the Year of Study variable which are proportions of the total migrant or non-migrant population.

⁎⁎ CPRD = Clinical Practice Research Datalink;.

⁎⁎⁎ Pyar = person years at risk.

In total, there were 25,112,116 all-cause consultations with a median of 11consultations per individual during the study period. Amongst the SRHR outcomes, events were highest for cervical screening (1274,612) and domestic violence and abuse was the least recorded outcome (9533).

**Fig. 2:**
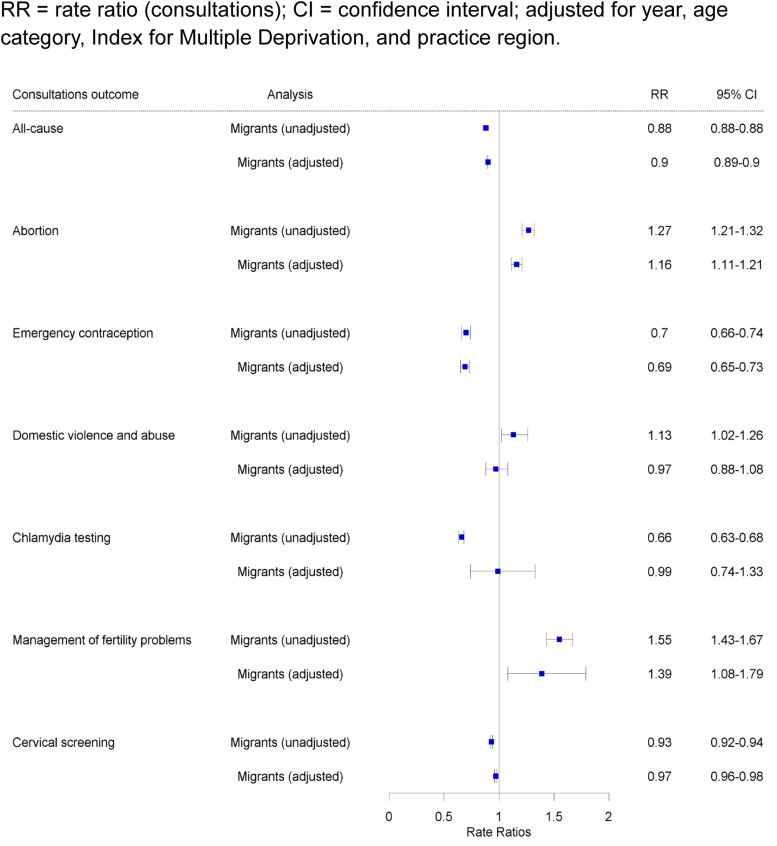
Rate ratios for all-cause consultations and SRHR consultations in migrant women versus non-migrant women in CPRD GOLD (2009–2018).

Fig. 2 shows an overview of the unadjusted and adjusted RRs of all consultation rate outcomes in migrant women compared to non-migrant women. After multivariable adjustment, rates of all-cause consultations in migrants (509 per 100 pyar) compared to non-migrants (583 per 100 pyar) were found to be significantly lower (RR 0.9; 95 % CI 0.9–0.9; *p*-value <0.001). Rates were also lower in migrants versus non-migrants for emergency contraception (0.58 per 100 pyar versus 0.83 per 100 pyar; RR 0.7; 95 % CI 0.7–0.7; *p*-value <0.001), and cervical screening (35.3 per 100 pyar versus 38.1 per 100 pyar; RR 0.96; 95 % CI 0.95–0.97; *p*-value <0.001). Higher rates of consultations were found in migrants versus non-migrants for abortion (1.24 per 100 pyar versus 1 per 100 pyar; RR 1.2; 95 % CI 1.1–1.2; *p*-value <0.001) and management of fertility problems (0.68 per 100 pyar versus 0.44 per 100; RR 1.39; 95 % CI 1.08–1.79; *p*-value <0.001). No significant difference was observed for chlamydia testing and domestic violence and abuse.

RR = rate ratio (consultations); CI = confidence interval; adjusted for year, age category, Index for Multiple Deprivation, and practice region.

These differences were impacted by sensitivity analysis of certainty of migration status and ethnicity (Appendix 2). The most notable findings were that the effect of higher rates for management of fertility problems disappeared for “definite” migrant group, all-cause consultations were higher not lower for migrants of Asian/Asian British (RR 1.14; 95 % CI 1.13–1.16) and Black/Black British ethnicity (RR 1.09; 95 % CI 1.07–1.10) and the effects observed for SRHR outcomes were restricted to specific ethnic groups, though with no specific pattern. Matching based on age and year of cohort showed that the lower rate ratios in migrants versus non-migrants were still observed for all-cause consultations (RR 0.85; 95 % CI 0.85–0.86), emergency contraception (RR 0.65; 95 % CI 0.60–0.70) and cervical screening (RR 0.89; 95 % CI 0.88–0.90) whilst all other effects were lost.

### LARC sub-cohorts

Overall, the proportion of non-migrants who had ever been prescribed any type of LARC (12.2 %; 135,047/1107,894) was almost twice as high as the proportion of migrants who had ever been prescribed LARC (6.91 %; 6728/97,364). The percentage of the population who had ever been prescribed LARC was higher amongst individuals with a greater person years at risk in both the migrant and non-migrant groups. The most frequently prescribed LARC in both groups was POI at 8114 prescriptions per 100,000 pyar in non-migrants and 2688 prescriptions per 100,000 pyar in migrants.

**Fig. 3:**
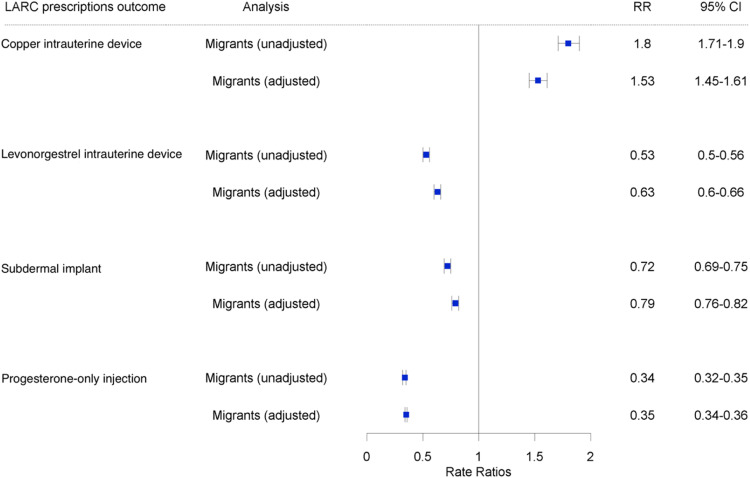
Rate ratios* for LARC prescriptions in migrant women aged 15–49 years old compared to non-migrant women (CPRD GOLD, 2009–2018).

Fig. 3 provides unadjusted and multivariable adjusted rate ratios for LARC prescriptions in migrants compared to non-migrants. After multivariable adjustment, Cu-IUD prescriptions were higher in migrant women compared to non-migrant women (RR 1.53; 95 % CI 1.45–1.61; *p*-value <0.001) as well as strong evidence that prescriptions were lower in migrants compared to non-migrants for all three types of hormonal LARC: LNG-IUD (RR 0.63; 95 % CI 0.60–0.66; *p*-value <0.001); SDI (RR 0.72; 95 % CI 0.69–0.75; *p*-value <0.001); POI (RR 0.35; 95 % CI 0.34–0.36; *p*-value <0.001). These effects were maintained when stratifying by certainty of migration, matching the cohort based on age and time of cohort entry, and using different estimates of pregnancy duration estimates. In our sensitivity analyses, ethnic grouping impacted evidence for differences in LARC prescription rates in migrants compared to non-migrants (see supplementary appendix 2). The most notable finding was a higher SDI prescription rate for the Asian/Asian British ethnic group (RR 1.17; 95 % CI 1.06–1.30; *p*-value <0.001) in migrants versus non-migrants.

*adjusted for age group, practice region, deprivation status, and year of study; LARC = long-acting reversible contraceptives; RR = rate ratio (prescriptions); CI = confidence interval

## Discussion

We found that rates of consultations in migrants compared to non-migrants were lower for all-causes, emergency contraception, and cervical screening and higher for abortion and management of fertility problems. No difference was observed for chlamydia testing and domestic violence and abuse. The effect of migration on outcomes varied across ethnic groups, but a consistent or predictable pattern did not emerge, emphasising the importance of not generalising across different migrant groups or considering them as a homogeneous group.

The proportion of non-migrants who had ever used LARC was almost twice as high as the proportion of migrants. In both groups, POI was the most prescribed LARC. After multivariable adjustment, the analysis found that Cu-IUD prescription rates were higher in migrant women compared to non-migrants and that prescription rates for hormonal LARCs were lower in migrants compared to non-migrants. This effect was maintained when stratifying by certainty of migration status, in a matched cohort based on age and time of cohort entry, and when assuming minimum pregnancy durations.

The study has several strengths. First, it is one of most comprehensive and representative of primary care datasets in England. Second, we used validated EHR codelists to define migration, ethnicity (Mathur et al., 2014) and SRHR outcomes. Third, we explicitly address heterogeneity in migration group by conducting subgroup analyses which considered how outcomes differ by migrants with different ethnic groups and certainty of migration status. Fourth, we created separate cohorts for each of the four LARC types with large sample sizes and included a wide range of conditions to determine cohort eligibility based on standard criteria for contraception eligibility (the UK MEC) (Faculty of Sexual and Reproductive Health 2019).

Our previous feasibility study showed that the migrant code list we used provides a large sample size and is nationally representative in relation to sex and geographical origin, but that recording of migrants is low compared to the proportion of migrants recorded by ONS (Pathak et al., 2021). As a result, a high proportion of migrants living in England were not identified by the codelist. If migrants are registering for primary care but do not having their migration status recorded there will be misclassification bias. Whilst migrants missed by the codelist could be a small percentage of the non-migrant population, they are likely to be a large percentage of the migrant group and therefore could change the migrant consultation rates. Recording of migration using this codelist is worse for age groups over 50 years old and better for the Asian/Asian British ethnic group compared to other ethnic groups. Our cohort has a large proportion younger age groups and therefore may overestimate outcomes associated with younger age groups, although the multivariate model used included adjusting for age group. The lack of representativeness of the codelist in terms of ethnicity also means caution must be applied when interpreting any findings as ethnicity may impact how migration is recorded. This is especially important for the present study where outcomes are known to be associated with ethnicity: white women are more likely to access cervical screening than women from other ethnic groups (Moser et al., 2009). Another important limitation of the codelist is that data describing the migration trajectory, such as timing, reason and region of migration, are not captured in EHRs. Bespoke linkages would be required to be able to explore the effect of these on SRHR outcomes and fully describe the heterogeneity within the migrant group. Finally, this study was completed before a method to define transgender and gender diverse individuals in CPRD was available. It has therefore not been possible in this study to address the needs of migrant individuals who were assigned female at birth and are no longer recorded as female in CPRD, but who may have overlapping needs such as cervical screening.

This study describes the rate of all-cause and SRHR consultations and LARC prescriptions in migrant women living in England and registered with primary care, thereby addressing an important research gap in the literature which has implications for policy and practice. Our findings suggest a difference in healthcare resource use between migrants and non-migrants and identify opportunities for tailored interventions including access to primary care, long-acting reversible contraceptives, emergency contraception and cervical screening. This builds on our previous study which showed that migrants in England were disproportionately impacted by the COVID-19 pandemic (Zhang et al., 2022). The World Health Organisation has produced guidelines and tools for stakeholders to move towards universal health coverage in SRHR with a particular focus on integrating comprehensive SRHR in primary care and monitoring and evaluation (World Health Organisation 2022). Our study demonstrates that it is possible to use English primary care electronic health records to adopt an inclusive approach to examining SRHR health needs. We recommend that the findings of this study are used to co-produce interventions and policy with migrant populations that can be evaluated using national electronic health records and actualise sexual and reproductive health as a human right.

## Data sharing

Our code lists used are freely available on GitHub (Benchimol et al., 2015).

## Funding

This work was supported by the 10.13039/100010269Wellcome Trust through a Clinical Research Career Development Fellowship to RWA [206602] and a Clinical Research Training Fellowship to NP [211162]. ACH's salary was provided by Central and North West London NHS Community Trust. SD is supported by an Alan Turing Fellowship and the UCL Hospitals Biomedical Research Centre (UCLH BRC). RM is supported by Barts Charity (MGU0504). The views expressed are those of the authors and not necessarily those of the funders or affiliated organisations.

## CRediT authorship contribution statement

**Neha Pathak:** Conceptualization, Funding acquisition, Methodology, Project administration, Writing – original draft, Writing – review & editing. **Claire X. Zhang:** Methodology, Writing – review & editing. **Yamina Boukari:** Methodology, Writing – review & editing. **Rachel Burns:** Methodology, Writing – review & editing. **Dee Menezes:** Methodology, Writing – review & editing. **Gregory Hugenholtz:** Methodology, Writing – review & editing. **Rebecca S French:** Methodology, Writing – review & editing. **Arturo Gonzalez-Izquierdo:** Methodology, Writing – review & editing. **Rohini Mathur:** Methodology, Writing – review & editing. **Spiros Denaxas:** Methodology, Writing – review & editing. **Andrew Hayward:** Conceptualization, Methodology, Supervision, Writing – review & editing. **Pam Sonnenberg:** Conceptualization, Methodology, Supervision, Writing – review & editing. **Robert W. Aldridge:** Conceptualization, Funding acquisition, Methodology, Supervision, Writing – original draft, Writing – review & editing.

## Declaration of competing interest

The authors declare the following financial interests/personal relationships which may be considered as potential competing interests: RM is part of the Genes & Health programme, which is part-funded (including salary contributions) by a Life Sciences Consortium comprising Astra Zeneca PLC, Bristol-Myers Squibb Company, GlaxoSmithKline Research and Development Limited, Maze Therapeutics Inc, Merck Sharp & Dohme LLC, Novo Nordisk A/S, Pfizer Inc, Takeda Development Centre Americas Inc. YB's spouse is employed by Elsevier as a Software Engineer

## Appendix Supplementary materials

Image, application 1

Image, application 2
