# Supplementary material for: Sexual and reproductive health and rights of migrant women attending primary care in England: A population-based cohort study of 1.2 million individuals of reproductive age (2009–2018)
Source: J Migr Health. 2024 Jan 17;9:100214. doi: 10.1016/j.jmh.2024.100214 (PMC10847991; doi:10.1016/j.jmh.2024.100214)
Supplement: Supplementary file 1 [file mmc1.docx]

###### Appendix 1. Final codelist to define migration and links to codelists to define SRHR outcomes + covariates used in the study

Table 1.1. International migration phenotype for EHR databases using the Read term system including CPRD GOLD

| Certainty of migration status | Type of code | Read code | Read term | Medcode |
| --- | --- | --- | --- | --- |
| Definite | Born outside of the UK | 13e..00 | Country of birth (Asian) | 11552 |
| Definite | Born outside of the UK | 13gf.00 | Born in South Africa | 12458 |
| Definite | Born outside of the UK | 13eG.00 | Born in Iraq | 12713 |
| Definite | Born outside of the UK | 13eH.00 | Born in Israel | 25007 |
| Definite | Born outside of the UK | 13go.00 | Born in Zimbabwe | 25008 |
| Definite | Born outside of the UK | 13eY.00 | Born in Philippines | 25092 |
| Definite | Born outside of the UK | 13gi.00 | Born in Tanzania | 25133 |
| Definite | Born outside of the UK | 13dl.00 | Born in Yugoslavia | 25256 |
| Definite | Born outside of the UK | 13gC.00 | Born in Congo | 25664 |
| Definite | Born outside of the UK | 13dM.00 | Born in Kosovo | 25730 |
| Definite | Born outside of the UK | 13eW.00 | Born in Pakistan | 25995 |
| Definite | Born outside of the UK | 13eo.00 | Born in Vietnam | 26334 |
| Definite | Born outside of the UK | 13eF.00 | Born in Iran | 26426 |
| Definite | Born outside of the UK | 13dA.00 | Born in Czech Republic | 26463 |
| Definite | Born outside of the UK | 13dP.00 | Born in Lithuania | 28301 |
| Definite | Born outside of the UK | 13eg.00 | Born in Syria | 28529 |
| Definite | Born outside of the UK | 13gY.00 | Born in Niger | 30224 |
| Definite | Born outside of the UK | 13f5.00 | Born in Canada | 30606 |
| Definite | Born outside of the UK | 13f..00 | Country of birth (American) | 32053 |
| Definite | Born outside of the UK | 13d0.00 | Born in Albania | 32055 |
| Definite | Born outside of the UK | 13e8.00 | Born in China | 32058 |
| Definite | Born outside of the UK | 13e0.00 | Born in Afghanistan | 32060 |
| Definite | Born outside of the UK | 13h0.00 | Born in Australia | 32061 |
| Definite | Born outside of the UK | 13jC.00 | Born in Trinidad and Tobago | 32062 |
| Definite | Born outside of the UK | 13gJ.00 | Born in Ghana | 32067 |
| Definite | Born outside of the UK | 13gl.00 | Born in Uganda | 32068 |
| Definite | Born outside of the UK | 13eI.00 | Born in Japan | 32070 |
| Definite | Born outside of the UK | 13fL.00 | Born in USA | 32072 |
| Definite | Born outside of the UK | 13h1.00 | Born in New Zealand | 32074 |
| Definite | Born outside of the UK | 13f3.00 | Born in Brazil | 32075 |
| Definite | Born outside of the UK | 13j6.00 | Born in Jamaica | 32076 |
| Definite | Born outside of the UK | 13g0.00 | Born in Algeria | 32079 |
| Definite | Born outside of the UK | 13dH.00 | Born in Greece | 32080 |
| Definite | Born outside of the UK | 13dF.00 | Born in France | 32081 |
| Definite | Born outside of the UK | 13eD.00 | Born in India | 32082 |
| Definite | Born outside of the UK | 13dG.00 | Born in Germany | 32085 |
| Definite | Born outside of the UK | 13j2.00 | Born in Barbados | 32089 |
| Definite | Born outside of the UK | 13fN.00 | Born in Venezuela | 32090 |
| Definite | Born outside of the UK | 13dW.00 | Born in Poland | 32094 |
| Definite | Born outside of the UK | 13de.00 | Born in Spain | 32097 |
| Definite | Born outside of the UK | 13df.00 | Born in Sweden | 32098 |
| Definite | Born outside of the UK | 13gM.00 | Born in Ivory Coast | 32099 |
| Definite | Born outside of the UK | 13g..00 | Country of birth (African) | 32102 |
| Definite | Born outside of the UK | 13di.00 | Born in Ukraine | 32103 |
| Definite | Born outside of the UK | 13gV.00 | Born in Morocco | 32108 |
| Definite | Born outside of the UK | 13gZ.00 | Born in Nigeria | 32111 |
| Definite | Born outside of the UK | 13gN.00 | Born in Kenya | 32112 |
| Definite | Born outside of the UK | 13dK.00 | Born in Ireland | 32113 |
| Definite | Born outside of the UK | 13ek.00 | Born in Turkey | 32114 |
| Definite | Born outside of the UK | 13gS.00 | Born in Malawi | 32115 |
| Definite | Born outside of the UK | 13dL.00 | Born in Italy | 32116 |
| Definite | Born outside of the UK | 13dc.00 | Born in Slovakia | 32119 |
| Definite | Born outside of the UK | 13fB.00 | Born in Grenada | 32120 |
| Definite | Born outside of the UK | 13e3.00 | Born in Bangladesh | 32125 |
| Definite | Born outside of the UK | 13eb.00 | Born in Russia | 32127 |
| Definite | Born outside of the UK | 13eM.00 | Born in Kyrgyzstan | 32128 |
| Definite | Born outside of the UK | 13j9.00 | Born in St. Lucia | 32131 |
| Definite | Born outside of the UK | 13ej.00 | Born in Thailand | 32135 |
| Definite | Born outside of the UK | 13dD.00 | Born in Estonia | 32139 |
| Definite | Born outside of the UK | 13gU.00 | Born in Mauritius | 32140 |
| Definite | Born outside of the UK | 13ec.00 | Born in Saudi Arabia | 32144 |
| Definite | Born outside of the UK | 13dh.00 | Born in The Netherlands | 32150 |
| Definite | Born outside of the UK | 13ed.00 | Born in Singapore | 32157 |
| Definite | Born outside of the UK | 13gG.00 | Born in Ethiopia | 32158 |
| Definite | Born outside of the UK | 13dX.00 | Born in Portugal | 32160 |
| Definite | Born outside of the UK | 13gW.00 | Born in Mozambique | 32162 |
| Definite | Born outside of the UK | 13fJ.00 | Born in Peru | 32166 |
| Definite | Born outside of the UK | 13g5.00 | Born in Burundi | 32167 |
| Definite | Born outside of the UK | 13gn.00 | Born in Zambia | 32168 |
| Definite | Born outside of the UK | 13d7.00 | Born in Bulgaria | 32169 |
| Definite | Born outside of the UK | 13eP.00 | Born in Malaysia | 32171 |
| Definite | Born outside of the UK | 13dE.00 | Born in Finland | 32173 |
| Definite | Born outside of the UK | 13dB.00 | Born in Denmark | 32186 |
| Definite | Born outside of the UK | 13ge.00 | Born in Somalia | 32189 |
| Definite | Born outside of the UK | 13d9.00 | Born in Cyprus | 32190 |
| Definite | Born outside of the UK | 13gd.00 | Born in Sierra Leone | 32197 |
| Definite | Born outside of the UK | 13fF.00 | Born in Mexico | 32201 |
| Definite | Born outside of the UK | 13e7.00 | Born in Chechnya | 32202 |
| Definite | Born outside of the UK | 13gI.00 | Born in Gambia | 32207 |
| Definite | Born outside of the UK | 13eT.00 | Born in Nepal | 32217 |
| Definite | Born outside of the UK | 13eC.00 | Born in Hong Kong | 32220 |
| Definite | Born outside of the UK | 13gX.00 | Born in Namibia | 32233 |
| Definite | Born outside of the UK | 13gP.00 | Born in Liberia | 32237 |
| Definite | Born outside of the UK | 13dN.00 | Born in Latvia | 32242 |
| Definite | Born outside of the UK | 13ef.00 | Born in Sri Lanka | 32245 |
| Definite | Born outside of the UK | 13e6.00 | Born in Burma | 32254 |
| Definite | Born outside of the UK | 13g7.00 | Born in Cameroon | 32255 |
| Definite | Born outside of the UK | 13g1.00 | Born in Angola | 32260 |
| Definite | Born outside of the UK | 13e2.00 | Born in Bahrain | 32273 |
| Definite | Born outside of the UK | 13k4.00 | Born in Seychelles | 32293 |
| Definite | Born outside of the UK | 13j0.00 | Born in Antigua and Barbuda | 32301 |
| Definite | Born outside of the UK | 13f7.00 | Born in Columbia | 32303 |
| Definite | Born outside of the UK | 13jB.00 | Born in Togo | 32304 |
| Definite | Born outside of the UK | 13gc.00 | Born in Senegal | 32309 |
| Definite | Born outside of the UK | 13f9.00 | Born in Ecuador | 32311 |
| Definite | Born outside of the UK | 13d2.00 | Born in Austria | 32313 |
| Definite | Born outside of the UK | 13f0.00 | Born in Argentina | 32325 |
| Definite | Born outside of the UK | 13ga.00 | Born in Rwanda | 32331 |
| Definite | Born outside of the UK | 13gE.00 | Born in Egypt | 32333 |
| Definite | Born outside of the UK | 13f4.00 | Born in British Guyana | 32342 |
| Definite | Born outside of the UK | 13dZ.00 | Born in Romania | 32345 |
| Definite | Born outside of the UK | 13gL.00 | Born in Guinea Republic | 32347 |
| Definite | Born outside of the UK | 13d4.00 | Born in Belgium | 32352 |
| Definite | Born outside of the UK | 13e1.00 | Born in Armenia | 32361 |
| Definite | Born outside of the UK | 13j4.00 | Born in Dominican Republic | 32369 |
| Definite | Born outside of the UK | 13eh.00 | Born in Taiwan | 32390 |
| Definite | Born outside of the UK | 13d6.00 | Born in Bosnia - Herzegovnia | 32397 |
| Definite | Born outside of the UK | 13k..00 | Country of birth (Pacific) | 32417 |
| Definite | Born outside of the UK | 13dI.00 | Born in Hungary | 32688 |
| Definite | Born outside of the UK | 13gk.00 | Born in Tunisia | 32741 |
| Definite | Born outside of the UK | 13dg.00 | Born in Switzerland | 32807 |
| Definite | Born outside of the UK | 13gg.00 | Born in Sudan | 36794 |
| Definite | Born outside of the UK | 13gR.00 | Born in Madagascar | 37197 |
| Definite | Born outside of the UK | 13fD.00 | Born in Guyana | 38075 |
| Definite | Born outside of the UK | 13gA.00 | Born in Chad | 38117 |
| Definite | Born outside of the UK | 13dS.00 | Born in Moldavia | 39974 |
| Definite | Born outside of the UK | 13gm.00 | Born in Zaire | 41209 |
| Definite | Born outside of the UK | 13d8.00 | Born in Croatia | 41210 |
| Definite | Born outside of the UK | 13eO.00 | Born in Lebanon | 41211 |
| Definite | Born outside of the UK | 13dV.00 | Born in Norway | 41213 |
| Definite | Born outside of the UK | 13eL.00 | Born in Kuwait | 41217 |
| Definite | Born outside of the UK | 13ee.00 | Born in South Korea | 41228 |
| Definite | Born outside of the UK | 13f2.00 | Born in Bolivia | 41230 |
| Definite | Born outside of the UK | 13d5.00 | Born in Belorussia | 41233 |
| Definite | Born outside of the UK | 13fE.00 | Born in Honduras | 41280 |
| Definite | Born outside of the UK | 13eE.00 | Born in Indonesia | 41289 |
| Definite | Born outside of the UK | 13j3.00 | Born in Cuba | 41290 |
| Definite | Born outside of the UK | 13f6.00 | Born in Chile | 41291 |
| Definite | Born outside of the UK | 13eK.00 | Born in Kazakhstan | 41292 |
| Definite | Born outside of the UK | 13eX.00 | Born in Palestine | 41297 |
| Definite | Born outside of the UK | 13el.00 | Born in Turkmenistan | 41302 |
| Definite | Born outside of the UK | 13dd.00 | Born in Slovenia | 41304 |
| Definite | Born outside of the UK | 13eJ.00 | Born in Jordan | 41311 |
| Definite | Born outside of the UK | 13dJ.00 | Born in Iceland | 41312 |
| Definite | Born outside of the UK | 13g3.00 | Born in Botswana | 41316 |
| Definite | Born outside of the UK | 13g2.00 | Born in Benin | 41318 |
| Definite | Born outside of the UK | 13h..00 | Country of birth (Australasian) | 41327 |
| Definite | Born outside of the UK | 13d3.00 | Born in Azerbaijan | 41337 |
| Definite | Born outside of the UK | 13gQ.00 | Born in Libya | 41341 |
| Definite | Born outside of the UK | 13ep.00 | Born in Yemen | 41344 |
| Definite | Born outside of the UK | 13gh.00 | Born in Swaziland | 41350 |
| Definite | Born outside of the UK | 13eS.00 | Born in Mongolia | 41351 |
| Definite | Born outside of the UK | 13e9.00 | Born in Democratic People’s Republic of Korea | 41354 |
| Definite | Born outside of the UK | 13ea.00 | Born in Republic of Korea | 41356 |
| Definite | Born outside of the UK | 13dY.00 | Born in Republic of Ireland | 41357 |
| Definite | Born outside of the UK | 13dR.00 | Born in Malta | 41364 |
| Definite | Born outside of the UK | 13gK.00 | Born in Guinea Bissau | 41365 |
| Definite | Born outside of the UK | 13fM.00 | Born in Uruguay | 41367 |
| Definite | Born outside of the UK | 13em.00 | Born in United Arab Emirates | 41372 |
| Definite | Born outside of the UK | 13fI.00 | Born in Paraguay | 41399 |
| Definite | Born outside of the UK | 13en.00 | Born in Uzbekistan | 41402 |
| Definite | Born outside of the UK | 13k0.00 | Born in Fiji | 42635 |
| Definite | Born outside of the UK | 13eZ.00 | Born in Qatar | 42639 |
| Definite | Born outside of the UK | 13k6.00 | Born in Tonga | 47559 |
| Definite | Born outside of the UK | 13k5.00 | Born in Solomon Islands | 48297 |
| Definite | Born outside of the UK | 13eB.00 | Born in Georgia | 49402 |
| Definite | Born outside of the UK | 13j..00 | Country of birth (Atlantic) | 49907 |
| Definite | Born outside of the UK | 13eV.00 | Born in Oman | 51778 |
| Definite | Born outside of the UK | 13eA.00 | Born in East Timor | 57186 |
| Definite | Born outside of the UK | 13k7.00 | Born in Tuvalu | 57189 |
| Definite | Born outside of the UK | 13j5.00 | Born in Haiti | 58192 |
| Definite | Born outside of the UK | 13g4.00 | Born in Burkina Faso | 58527 |
| Definite | Born outside of the UK | 13j1.00 | Born in Bahamas | 58533 |
| Definite | Born outside of the UK | 13gH.00 | Born in Gabon | 59657 |
| Definite | Born outside of the UK | 13e5.00 | Born in Brunei | 62298 |
| Definite | Born outside of the UK | 13fH.00 | Born in Panama | 63923 |
| Definite | Born outside of the UK | 13gj.00 | Born in The Gambia | 63927 |
| Definite | Born outside of the UK | 13fA.00 | Born in El Salvador | 63943 |
| Definite | Born outside of the UK | 13k3.00 | Born in Papua New Guinea | 64120 |
| Definite | Born outside of the UK | 13gp.00 | Born in Eritrea | 64949 |
| Definite | Born outside of the UK | 13f8.00 | Born in Costa Rica | 64984 |
| Definite | Born outside of the UK | 13eR.00 | Born in Mali | 65310 |
| Definite | Born outside of the UK | 13jA.00 | Born in St. Vincent | 66551 |
| Definite | Born outside of the UK | 13j8.00 | Born in St. Kitts and Nevis | 66553 |
| Definite | Born outside of the UK | 13eU.00 | Born in North Korea | 66560 |
| Definite | Born outside of the UK | 13gD.00 | Born in Djibouti | 68866 |
| Definite | Born outside of the UK | 13dQ.00 | Born in Luxembourg | 69131 |
| Definite | Born outside of the UK | 13j7.00 | Born in Puerto Rico | 69135 |
| Definite | Born outside of the UK | 13gT.00 | Born in Mauritania | 69143 |
| Definite | Born outside of the UK | 13fK.00 | Born in Suriname | 69426 |
| Definite | Born outside of the UK | 13eN.00 | Born in Laos | 69431 |
| Definite | Born outside of the UK | 13g8.00 | Born in Cape Verde Islands | 69560 |
| Definite | Born outside of the UK | 13fC.00 | Born in Guatemala | 69806 |
| Definite | Born outside of the UK | 13dT.00 | Born in Monaco | 71190 |
| Definite | Born outside of the UK | 13fG.00 | Born in Nicaragua | 74892 |
| Definite | Born outside of the UK | 13eQ.00 | Born in Maldives | 91328 |
| Definite | Born outside of the UK | 13gO.00 | Born in Lesotho | 93697 |
| Definite | Born outside of the UK | 13gF.00 | Born in Equatorial Guinea | 93923 |
| Definite | Born outside of the UK | 13d1.00 | Born in Andorra | 93935 |
| Definite | Born outside of the UK | 13f1.00 | Born in Belize | 94050 |
| Definite | Born outside of the UK | 13dO.00 | Born in Liechtenstein | 95708 |
| Definite | Born outside of the UK | 13t..00 | Born in British overseas territory | 96295 |
| Definite | Born outside of the UK | 13g6.00 | Born in Cambodia | 96636 |
| Definite | Born outside of the UK | 13dm.00 | Born in former Yugoslav Republic of Macedonia | 96824 |
| Definite | Born outside of the UK | 13e4.00 | Born in Bhutan | 97390 |
| Definite | Born outside of the UK | 13t1.00 | Born in Bermuda | 98038 |
| Definite | Born outside of the UK | 13da.00 | Born in San Marino | 98530 |
| Definite | Born outside of the UK | 13dj.00 | Born in Vatican City | 99119 |
| Definite | Born outside of the UK | 13g9.00 | Born in Central African Republic | 99258 |
| Definite | Born outside of the UK | 13jD.00 | Born in Dominica | 99431 |
| Definite | Born outside of the UK | 13ei.00 | Born in Tajikistan | 100007 |
| Definite | Born outside of the UK | 13dn.00 | Born in Serbia | 100517 |
| Definite | Born outside of the UK | 13gB.00 | Born in Comoros Islands | 101158 |
| Definite | Born outside of the UK | 13gb.00 | Born in Sao Tome and Principe | 101591 |
| Definite | Born outside of the UK | 13Zq.00 | Country of birth unknown | 101846 |
| Definite | Born outside of the UK | 13v0.00 | Born in Martinique | 103364 |
| Definite | Born outside of the UK | 13k9.00 | Born in Western Samoa | 103965 |
| Definite | Born outside of the UK | 13t2.00 | Born in Anguilla | 104983 |
| Definite | Born outside of the UK | 13t0.00 | Born in Montserrat | 105923 |
| Definite | Born outside of the UK | 13jE.00 | Born in Aruba | 108271 |
| Definite | Born outside of the UK | 13v7.00 | Born in Guadeloupe | 108936 |
| Definite | Born outside of the UK | 13eq.00 | Born in Christmas Island | 109226 |
| Definite | Born outside of the UK | 13dq.00 | Born in Republic of Moldova | 109260 |
| Definite | Born outside of the UK | 13t5.00 | Born in Saint Helena, Ascension and Tristan da Cunha | 109276 |
| Definite | Born outside of the UK | 13do.00 | Born in Montenegro | 109457 |
| Definite | Born outside of the UK | 13jG.00 | Born in Saint Vincent and the Grenadines | 109458 |
| Definite | Born outside of the UK | 13t3.00 | Born in British Virgin Islands | 109727 |
| Definite | Born outside of the UK | 13kB.00 | Born in American Samoa | 109791 |
| Definite | Born outside of the UK | 13dp.00 | Born in Belarus | 109898 |
| Definite | Born outside of the UK | 13gq.00 | Born in Democratic Republic of Congo | 109992 |
| Definite | Born outside of the UK | 13v4.00 | Born in French Guiana | 111051 |
| Definite | Born outside of the UK | 13k8.00 | Born in Vanuatu | 111392 |
| Definite | Born outside of the UK | 13jH.00 | Born in Sint Maarten | 111700 |
| Definite | Born outside of the UK | 13dx.00 | Born in Aland Islands | 111774 |
| Definite | Born outside of the UK | 13t9.00 | Born in Cayman Islands | 112269 |
| Definite | Visa status indicating migration | 13ZC.00 | Immigrant | 4114 |
| Definite | Visa status indicating migration | ZV70314 | [V]Immigration medical | 8929 |
| Definite | Visa status indicating migration | 133L.00 | Immigrant | 9292 |
| Definite | Visa status indicating migration | 13ZN.00 | Asylum seeker | 9627 |
| Definite | Visa status indicating migration | 13D3.00 | Social migrant | 23398 |
| Definite | Visa status indicating migration | 13ZB.00 | Refugee | 24403 |
| Definite | Visa status indicating migration | ZV70516 | [V]Refugee health examination | 25632 |
| Definite | Visa status indicating migration | 13D4.00 | Illegal migrant | 44407 |
| Definite | Visa status indicating migration | 133Q.00 | Family reunion immigrant | 47073 |
| Definite | Visa status indicating migration | 912H.00 | Overseas visitor | 47513 |
| Definite | Visa status indicating migration | 6951 | Immigration examination | 48029 |
| Definite | Visa status indicating migration | 69D8.00 | Exam. of refugee | 65503 |
| Definite | Visa status indicating migration | 13Zd.00 | Failed asylum seeker | 94906 |
| Definite | Visa status indicating migration | 13Zw.00 | Has United Kingdom student visa | 104123 |
| Definite | Visa status indicating migration | 918z.00 | Has United Kingdom general visitor visa | 107572 |
| Probable | First/main language not English | 13lZ.00 | Main spoken language Turkish | 22294 |
| Probable | First/main language not English | 041E.11 | Language interpreter | 22515 |
| Probable | First/main language not English | 13Z6000 | English as a second language | 23523 |
| Probable | First/main language not English | 13Z6500 | Language Punjabi | 24295 |
| Probable | First/main language not English | 13Z6300 | Language Hindi | 24296 |
| Probable | First/main language not English | 13Z6600 | Language Urdu | 24691 |
| Probable | First/main language not English | 13Z6200 | Language Gujurati | 24712 |
| Probable | First/main language not English | 13Z6100 | Language Bengali | 24741 |
| Probable | First/main language not English | 13lC.00 | Main spoken language Polish | 24881 |
| Probable | First/main language not English | 13b0.00 | Vietnamese language | 25410 |
| Probable | First/main language not English | 13lS.00 | Main spoken language Albanian | 25423 |
| Probable | First/main language not English | 13l2.00 | Main spoken language Cantonese | 25472 |
| Probable | First/main language not English | 13lx.00 | Main spoken language Thai | 25609 |
| Probable | First/main language not English | 13lp.00 | Main spoken language Malayalam | 25616 |
| Probable | First/main language not English | 13l5.00 | Main spoken language French | 25665 |
| Probable | First/main language not English | 13l1.00 | Main spoken language Bengali | 25802 |
| Probable | First/main language not English | 13lE.00 | Main spoken language Punjabi | 25829 |
| Probable | First/main language not English | 13lP.00 | Main spoken language Shona | 26078 |
| Probable | First/main language not English | 13Z6400 | Language Pashtu | 26196 |
| Probable | First/main language not English | 13lH.00 | Main spoken language Spanish | 26247 |
| Probable | First/main language not English | 13lb.00 | Main spoken language Vietnamese | 26335 |
| Probable | First/main language not English | 13l0.00 | Main spoken language Arabic | 26337 |
| Probable | First/main language not English | 13lL.00 | Main spoken language Urdu | 26361 |
| Probable | First/main language not English | 13l3.00 | Main spoken language Czech | 26464 |
| Probable | First/main language not English | 13lF.00 | Main spoken language Russian | 32427 |
| Probable | First/main language not English | 13lG.00 | Main spoken language Somali | 32456 |
| Probable | First/main language not English | 13lB.00 | Main spoken language Mandarin | 32776 |
| Probable | First/main language not English | 13lW.00 | Main spoken language Japanese | 36852 |
| Probable | First/main language not English | 13lt.00 | Main spoken language Serbian | 36862 |
| Probable | First/main language not English | 13lQ.00 | Main spoken language Italian | 36980 |
| Probable | First/main language not English | 13lN.00 | Main spoken language Kurdish | 46014 |
| Probable | First/main language not English | 13l8.00 | Main spoken language Hindi | 46029 |
| Probable | First/main language not English | 13lY.00 | Main spoken language Lithuanian | 46325 |
| Probable | First/main language not English | 13lK.00 | Main spoken language Tamil | 46861 |
| Probable | First/main language not English | 13lV.00 | Main spoken language Greek | 46974 |
| Probable | First/main language not English | 13li.00 | Main spoken language French Creole | 47007 |
| Probable | First/main language not English | 13lO.00 | Main spoken language Farsi | 47029 |
| Probable | First/main language not English | 13lJ.00 | Main spoken language Sylheti | 47627 |
| Probable | First/main language not English | 13ld.00 | Main spoken language Amharic | 47628 |
| Probable | First/main language not English | 13lR.00 | Main spoken language German | 47630 |
| Probable | First/main language not English | 13lw.00 | Main spoken language Tagalog | 47631 |
| Probable | First/main language not English | 13lI.00 | Main spoken language Swahili | 47641 |
| Probable | First/main language not English | 13lu.00 | Main spoken language Sinhala | 47643 |
| Probable | First/main language not English | 13lm.00 | Main spoken language Igbo | 47646 |
| Probable | First/main language not English | 13l6.00 | Main spoken language Gujerati | 48002 |
| Probable | First/main language not English | 13b4.00 | Mirpuri language | 52200 |
| Probable | First/main language not English | 13lM.00 | Main spoken language Yoruba | 54409 |
| Probable | First/main language not English | 13lT.00 | Main spoken language Croatian | 54410 |
| Probable | First/main language not English | 13lc.00 | Main spoken language Akan | 54413 |
| Probable | First/main language not English | 13lf.00 | Main spoken language Dutch | 54414 |
| Probable | First/main language not English | 13lX.00 | Main spoken language Korean | 54415 |
| Probable | First/main language not English | 13l9.00 | Main spoken language Iba | 54416 |
| Probable | First/main language not English | 13lv.00 | Main spoken language Swedish | 54417 |
| Probable | First/main language not English | 13lh.00 | Main spoken language Flemish | 56879 |
| Probable | First/main language not English | 13b3.00 | Creole language | 57462 |
| Probable | First/main language not English | 13ll.00 | Main spoken language Hebrew | 57755 |
| Probable | First/main language not English | 13lq.00 | Main spoken language Norwegian | 57758 |
| Probable | First/main language not English | 13l7.00 | Main spoken language Hausa | 58193 |
| Probable | First/main language not English | 13lr.00 | Main spoken language Pashto | 58643 |
| Probable | First/main language not English | 13sA.00 | English as a second language | 63932 |
| Probable | First/main language not English | 13ls.00 | Main spoken language Patois | 64948 |
| Probable | First/main language not English | 13la.00 | Main spoken language Ukrainian | 66685 |
| Probable | First/main language not English | 13lg.00 | Main spoken language Ethiopian | 69139 |
| Probable | First/main language not English | 13lo.00 | Main spoken language Luganda | 69153 |
| Probable | First/main language not English | 13lA.00 | Main spoken language Kutchi | 72379 |
| Probable | First/main language not English | 13lk.00 | Main spoken language Hakka | 91422 |
| Probable | First/main language not English | ZV60012 | [V]Social migrant | 93643 |
| Probable | First/main language not English | 13u0.00 | Main spoken language Bulgarian | 95897 |
| Probable | First/main language not English | 13ur.00 | Main spoken language Latvian | 95940 |
| Probable | First/main language not English | 13lT.11 | Main spoken language Serbo-Croatian | 95968 |
| Probable | First/main language not English | 13lt.11 | Main spoken language Serbo-Croatian | 95969 |
| Probable | First/main language not English | 13lO.11 | Main spoken language Persian | 95970 |
| Probable | First/main language not English | 13lu.11 | Main spoken language Sinhalese | 95974 |
| Probable | First/main language not English | 13w1.00 | Main spoken language Nepali | 95978 |
| Probable | First/main language not English | 13ua.00 | Main spoken language Hungarian | 96041 |
| Probable | First/main language not English | 13lE.11 | Main spoken language Panjabi | 96147 |
| Probable | First/main language not English | 13u5.00 | Main spoken language Afrikaans | 96148 |
| Probable | First/main language not English | 13u1.00 | Main spoken language Romanian | 96152 |
| Probable | First/main language not English | 13wL.00 | Main spoken language Telugu | 96163 |
| Probable | First/main language not English | 13wR.00 | Main spoken language Twi | 96223 |
| Probable | First/main language not English | 13wG.00 | Main spoken language Slovenian | 96230 |
| Probable | First/main language not English | 13wD.00 | Main spoken language Sindhi | 96240 |
| Probable | First/main language not English | 13ux.00 | Main spoken language Marathi | 96267 |
| Probable | First/main language not English | 13uj.00 | Main spoken language Kannada | 96268 |
| Probable | First/main language not English | 13uN.00 | Main spoken language Danish | 96289 |
| Probable | First/main language not English | 13u6.00 | Main spoken language Armenian | 96290 |
| Probable | First/main language not English | 13w5.00 | Main spoken language Quechua | 96317 |
| Probable | First/main language not English | 13uv.00 | Main spoken language Maltese | 96370 |
| Probable | First/main language not English | 13uu.00 | Main spoken language Malay | 96376 |
| Probable | First/main language not English | 13le.00 | Main spoken language Brawa | 96485 |
| Probable | First/main language not English | 13wM.00 | Main spoken language Tibetan | 96558 |
| Probable | First/main language not English | 13uG.00 | Main spoken language Burmese | 96559 |
| Probable | First/main language not English | 13uT.00 | Main spoken language Finnish | 96560 |
| Probable | First/main language not English | 13us.00 | Main spoken language Macedonian | 96611 |
| Probable | First/main language not English | 13wN.00 | Main spoken language Tongan | 96634 |
| Probable | First/main language not English | 13wT.00 | Main spoken language Uzbek | 96784 |
| Probable | First/main language not English | 13u2.00 | Main spoken language Oromo | 96857 |
| Probable | First/main language not English | 13u4.00 | Main spoken language Afar | 96868 |
| Probable | First/main language not English | 13wa.00 | Main spoken language Zulu | 96873 |
| Probable | First/main language not English | 13uQ.00 | Main spoken language Estonian | 96928 |
| Probable | First/main language not English | 13uk.00 | Main spoken language Kashmiri | 97015 |
| Probable | First/main language not English | 13uz.00 | Main spoken language Mongolian | 97038 |
| Probable | First/main language not English | 13wW.00 | Main spoken language Wolof | 97039 |
| Probable | First/main language not English | 13wQ.00 | Main spoken language Turkmen | 97041 |
| Probable | First/main language not English | 13u9.00 | Main spoken language Azerbaijani | 97083 |
| Probable | First/main language not English | 13wH.00 | Main spoken language Sundanese | 97131 |
| Probable | First/main language not English | 13uX.00 | Main spoken language Georgian | 97212 |
| Probable | First/main language not English | 13wX.00 | Main spoken language Xhosa | 97273 |
| Probable | First/main language not English | 13uc.00 | Main spoken language Indonesian | 97274 |
| Probable | First/main language not English | 13w3.00 | Main spoken language Oriya | 97297 |
| Probable | First/main language not English | 9NUC.11 | Persian language interpreter needed | 97298 |
| Probable | First/main language not English | 13wB.00 | Main spoken language Southern Sotho | 97439 |
| Probable | First/main language not English | 13w6.00 | Main spoken language Romansh | 97440 |
| Probable | First/main language not English | 13uB.00 | Main spoken language Basque | 97574 |
| Probable | First/main language not English | 13ul.00 | Main spoken language Kazakh | 97595 |
| Probable | First/main language not English | 9NUz.00 | Bulgarian language interpreter needed | 97644 |
| Probable | First/main language not English | 13wP.00 | Main spoken language Tsonga | 97685 |
| Probable | First/main language not English | 13uK.00 | Main spoken language Catalan | 97997 |
| Probable | First/main language not English | 9Nmm.00 | Burmese language interpreter needed | 98062 |
| Probable | First/main language not English | 13uZ.00 | Main spoken language Guarani | 98070 |
| Probable | First/main language not English | 13up.00 | Main spoken language Lao | 98132 |
| Probable | First/main language not English | 13um.00 | Main spoken language Kinyarwanda | 98194 |
| Probable | First/main language not English | 13uy.00 | Main spoken language Moldavian | 98215 |
| Probable | First/main language not English | 13uw.00 | Main spoken language Maori | 98255 |
| Probable | First/main language not English | 13w4.00 | Main spoken language Filipino | 98285 |
| Probable | First/main language not English | 13uY.00 | Main spoken language Kalaallisut | 98510 |
| Probable | First/main language not English | 13ub.00 | Main spoken language Icelandic | 98604 |
| Probable | First/main language not English | 13u8.00 | Main spoken language Aymara | 98762 |
| Probable | First/main language not English | 9NmQ.00 | Hungarian language interpreter needed | 98809 |
| Probable | First/main language not English | 9NUy.00 | Romanian language interpreter needed | 98841 |
| Probable | First/main language not English | 13wS.00 | Main spoken language Uighur | 99712 |
| Probable | First/main language not English | 9Nn1.00 | Tsonga language interpreter needed | 99794 |
| Probable | First/main language not English | 13ui.00 | Main spoken language Javanese | 100011 |
| Probable | First/main language not English | 13uL.00 | Main spoken language Slovak | 100013 |
| Probable | First/main language not English | 9NmA.00 | Macedonian language interpreter needed | 100438 |
| Probable | First/main language not English | 13uR.00 | Main spoken language Faeroese | 100707 |
| Probable | First/main language not English | 13uS.00 | Main spoken language Fijian | 100714 |
| Probable | First/main language not English | 13ug.00 | Main spoken language Inuktitut | 100716 |
| Probable | First/main language not English | 13uW.00 | Main spoken language Galician | 100743 |
| Probable | First/main language not English | 9Nn7.00 | Slovenian language interpreter needed | 100759 |
| Probable | First/main language not English | 13uH.00 | Main spoken language Belarusian | 100828 |
| Probable | First/main language not English | 13uD.00 | Main spoken language Bihari | 100949 |
| Probable | First/main language not English | 13wb.00 | Main spoken language Konkani | 101038 |
| Probable | First/main language not English | 13uJ.00 | Main spoken language Central Khmer | 101189 |
| Probable | First/main language not English | 13wA.00 | Main spoken language Dari | 101220 |
| Probable | First/main language not English | 9Nn4.00 | Telugu language interpreter needed | 101614 |
| Probable | First/main language not English | 13ut.00 | Main spoken language Malagasy | 101620 |
| Probable | First/main language not English | 13ud.00 | Main spoken language Interlingua | 101659 |
| Probable | First/main language not English | 13l9.11 | Main spoken language Iban | 101761 |
| Probable | First/main language not English | 13uM.00 | Main spoken language Corsican | 101788 |
| Probable | First/main language not English | 9NUc.11 | Punjabi language interpreter needed | 101814 |
| Probable | First/main language not English | 13uY.11 | Main spoken language Greenlandic | 102007 |
| Probable | First/main language not English | 13uC.00 | Main spoken language Dzongkha | 102127 |
| Probable | First/main language not English | 13uF.00 | Main spoken language Breton | 102128 |
| Probable | First/main language not English | 13ue.00 | Main spoken language Interlingue | 102129 |
| Probable | First/main language not English | 13u3.00 | Main spoken language Abkhazian | 102184 |
| Probable | First/main language not English | 13u7.00 | Main spoken language Assamese | 102218 |
| Probable | First/main language not English | 13uA.00 | Main spoken language Bashkir | 102259 |
| Probable | First/main language not English | 13wC.00 | Main spoken language Tswana | 102877 |
| Probable | First/main language not English | 13uV.00 | Main spoken language Frisian | 103200 |
| Probable | First/main language not English | 13w7.00 | Main spoken language Samoan | 103219 |
| Probable | First/main language not English | 13uq.00 | Main spoken language Bamun | 104071 |
| Probable | First/main language not English | 9NmC.00 | Latvian language interpreter needed | 104635 |
| Probable | First/main language not English | 13wE.00 | Main spoken language Ndebele | 104886 |
| Probable | First/main language not English | 9Nm6.00 | Brawa language interpreter needed | 104901 |
| Probable | First/main language not English | 9Nmd.00 | Catalan language interpreter needed | 105079 |
| Probable | First/main language not English | 13wV.00 | Main spoken language Tetum | 105523 |
| Probable | First/main language not English | 13wc.00 | Main spoken language Aragonese | 105529 |
| Probable | First/main language not English | 13wJ.00 | Main spoken language Tajik | 105960 |
| Probable | First/main language not English | 13Z6900 | First language not English | 108098 |
| Probable | First/main language not English | 9Nn6.00 | Turkmen language interpreter needed | 108184 |
| Probable | First/main language not English | 9NmE.00 | Kinyarwanda language interpreter needed | 109489 |
| Probable | First/main language not English | 13l6.11 | Main spoken language Gujarati | 109896 |
| Probable | First/main language not English | 13wF.00 | Main spoken language Swati | 111709 |
| Probable | First/main language not English | 13w0.00 | Main spoken language Nauru | 111734 |
| Probable | First/main language not English | 9Nm1.00 | Moldavian language interpreter needed | 111788 |
| Probable | First/main language not English | 13wZ.00 | Main spoken language Zhuang | 111789 |
| Probable | First/main language not English | 13wY.00 | Main spoken language Yiddish | 111874 |
| Probable | First/main language not English | 13w8.00 | Main spoken language Sango | 111919 |
| Probable | First/main language not English | 9NnC.00 | Sundanese language interpreter needed | 112222 |
| Probable | First/main language not English | 13uo.00 | Main spoken language Rundi | 112260 |
| Probable | First/main language not English | 13wK.00 | Main spoken language Tatar | 112321 |
| Probable | First/main language not English | 13lD.00 | Main spoken language Portuguese | 32728 |
| Possible | Non-UK origin | 1343 | Asian origin | 25801 |
| Possible | Non-UK origin | 1345 | South American origin | 32101 |
| Possible | Non-UK origin | 2263.11 | O/E - Asian origin | 32132 |
| Possible | Non-UK origin | 1344 | North American origin | 41150 |
| Possible | Non-UK origin | 1342 | African origin | 45125 |
| Possible | Non-UK origin | 134A.00 | West Indian origin | 45131 |
| Possible | Non-UK origin | 1347 | Indian origin | 45144 |
| Possible | Non-UK origin | 1348 | Middle Eastern origin | 47951 |
| Possible | Non-UK origin | 9SA5.00 | Other African countries (NMO) | 47969 |
| Possible | Non-UK origin | 1346 | Australian origin | 47975 |
| Possible | Non-UK origin | 2263 | O/E - Mongoloid origin | 66536 |

The full Read term codelists for all covariates and SRHR outcomes are held on the UCL Centre for Public Health Data Science GitHub and made freely available for public access.

1. Pathak N. CPRD GOLD migrant phenotype validation [Internet]. UCL-Public-Health-Data-Science; 2022 [cited 2022 Mar 1]. Available from: <https://github.com/UCL-Public-Health-Data-Science/CPRD-GOLD-migrant-phenotype-validation>
2. Pathak N. CPRD GOLD migrant sexual and reproductive health and rights (SRHR) [Internet]. UCL-Public-Health-Data-Science; 2022 [cited 2022 Mar 16]. Available from: https://github.com/UCL-Public-Health-Data-Science/CPRD-GOLD-migrant-sexual-and-reproductive-health-and-rights
