# Supplementary material for: Sexual and reproductive health and rights of migrant women attending primary care in England: A population-based cohort study of 1.2 million individuals of reproductive age (2009–2018)
Source: J Migr Health. 2024 Jan 17;9:100214. doi: 10.1016/j.jmh.2024.100214 (PMC10847991; doi:10.1016/j.jmh.2024.100214)

###### Appendix 2. Additional Figures – sensitivity analyses of certainty of migration status and ethnicity

Figure 2.1. Multivariable adjusted RRs for migrant women versus non-migrant women for all-cause consultation and SRHR-consultations stratified by certainty of migration status: main analysis and exact-matched sensitivity analysis results (CPRD GOLD, 2009-2018)


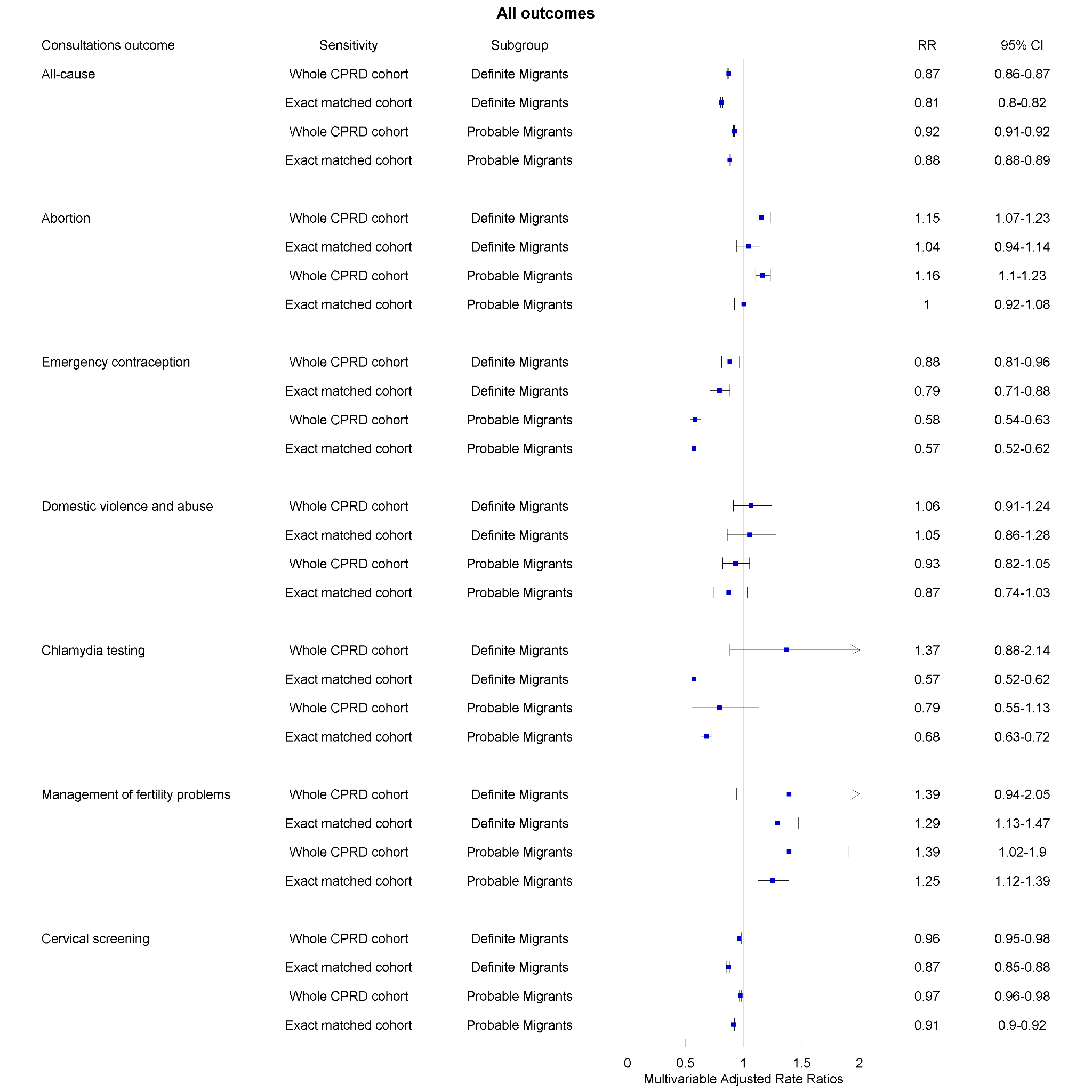


Figure 2.2 Multivariable adjusted RRs for all-cause and SRHR consultations in migrant women versus non-migrants stratified by ethnicity in the whole cohort and exact matched cohort (CPRD GOLD, 2009-2018)


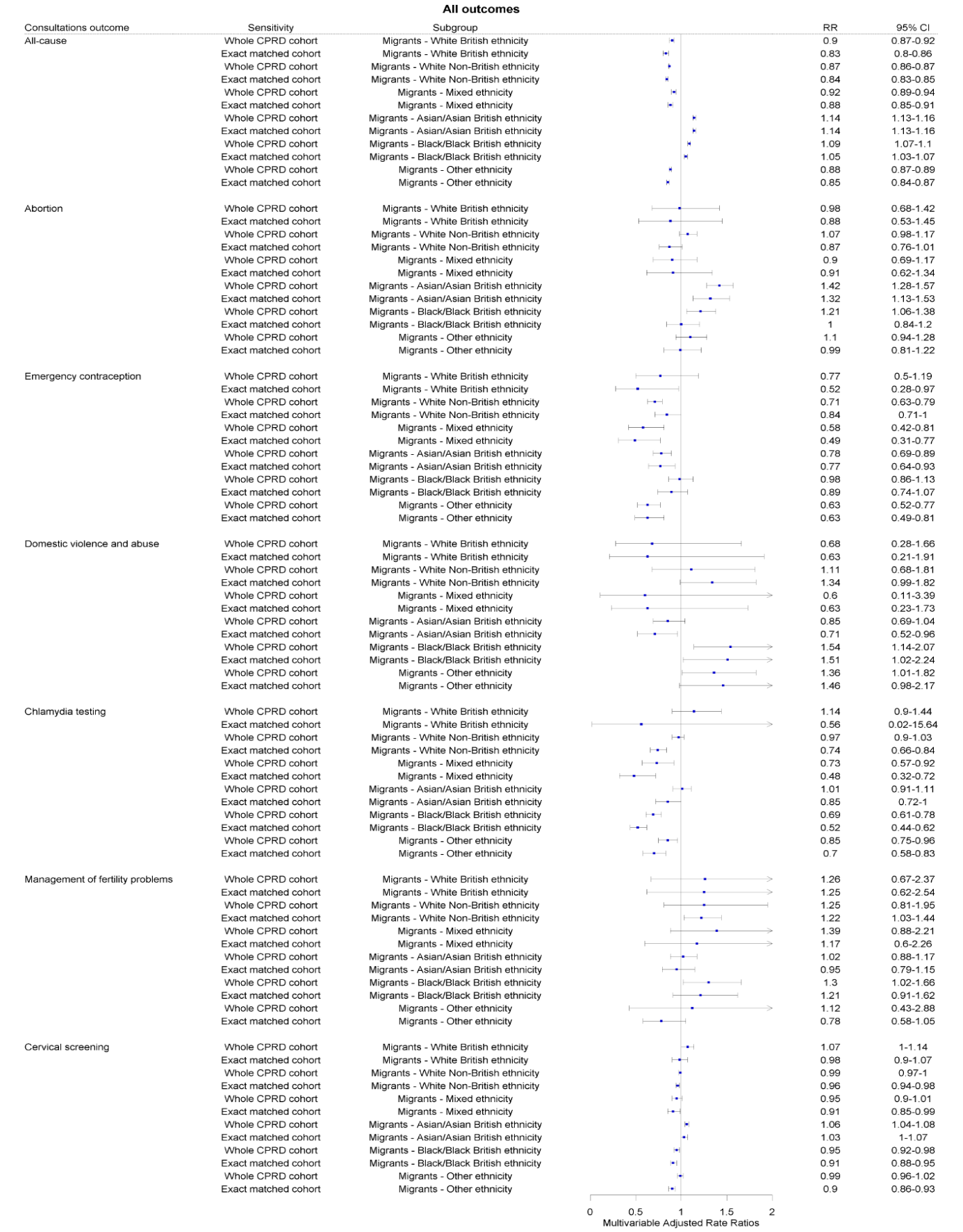


Figure 2.3. Multivariable adjusted rate ratios^†^ across subgroup and sensitivity analyses for Cu-IUD prescribing in migrant women compared to non-migrant women (CPRD GOLD, 2009-2018)

^†^=adjusted for age group, practice region, deprivation status, and year of study; RR = rate ratio; CI = confidence interval


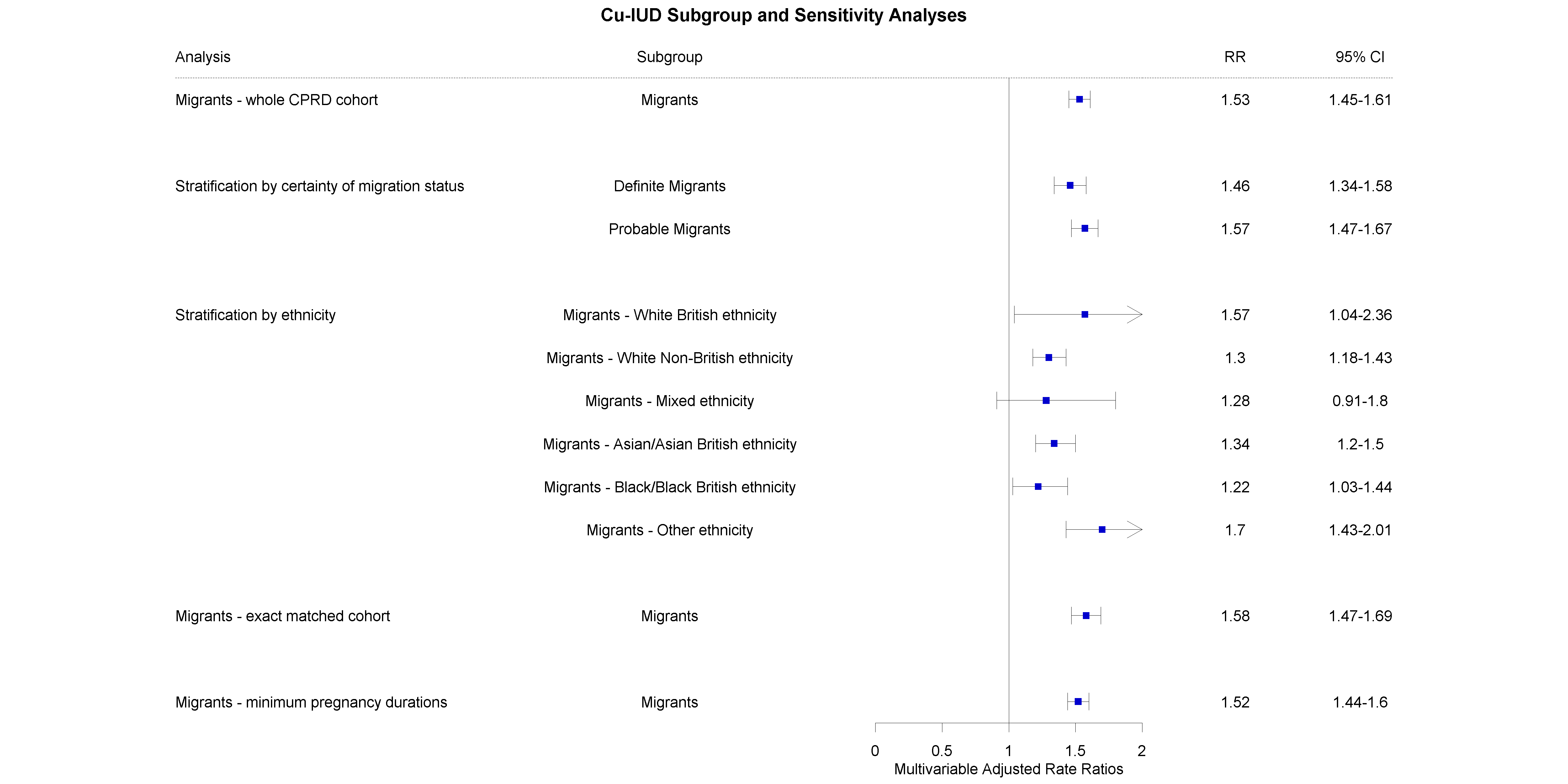


Figure 2.4 Multivariable adjusted rate ratios^†^ across subgroup and sensitivity analyses for LNG-IUD prescribing in migrant women compared to non-migrant women (CPRD GOLD, 2009-2018)

^†^=adjusted for age group, practice region, deprivation status, and year of study; RR = rate ratio; CI = confidence interval


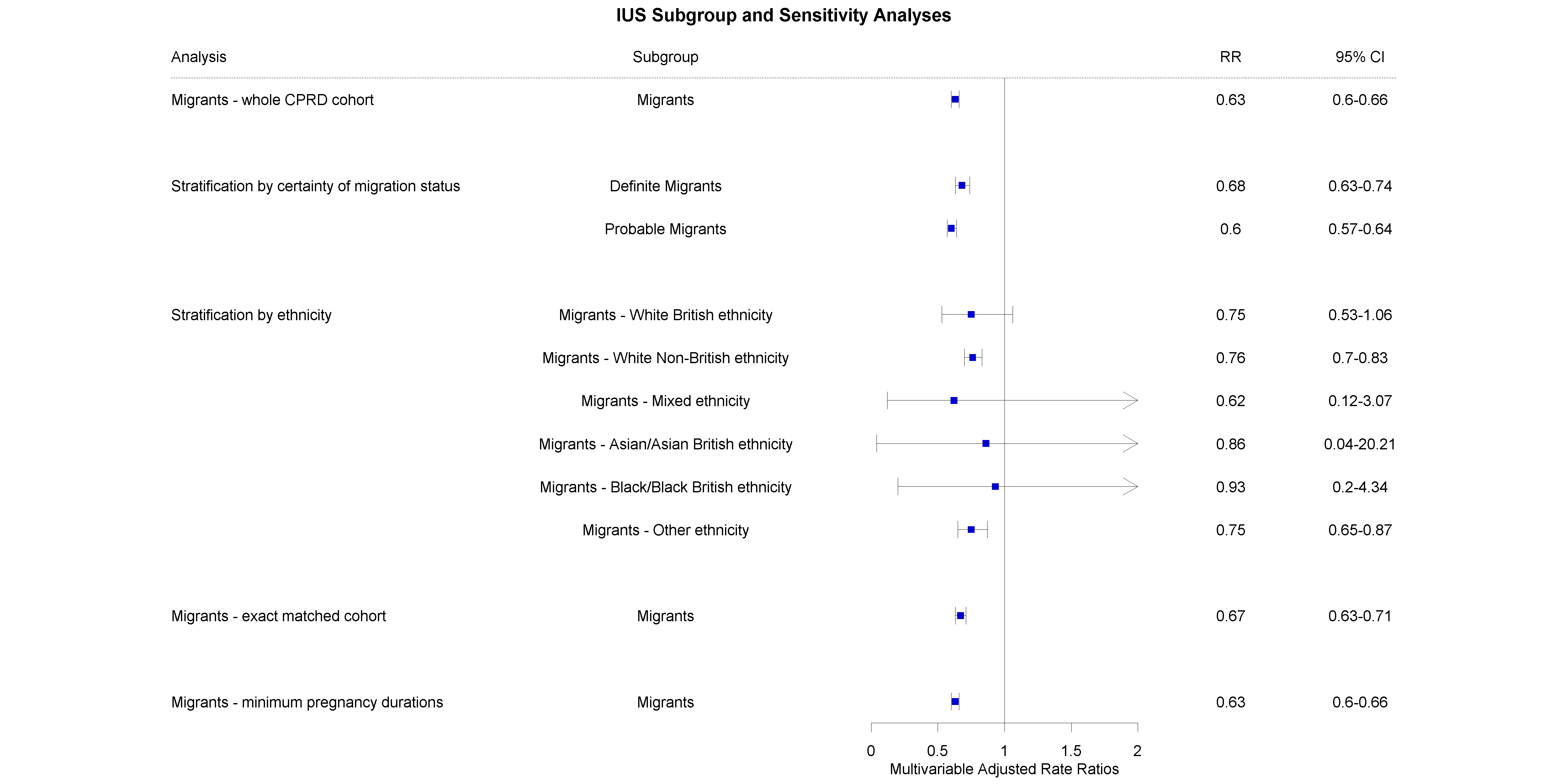


Figure 2.5. Multivariable adjusted rate ratios^†^ across subgroup and sensitivity analyses for SDI prescribing in migrant women compared to non-migrant women (CPRD GOLD, 2009-2018)

^†^=adjusted for age group, practice region, deprivation status, and year of study; RR = rate ratio; CI = confidence interval


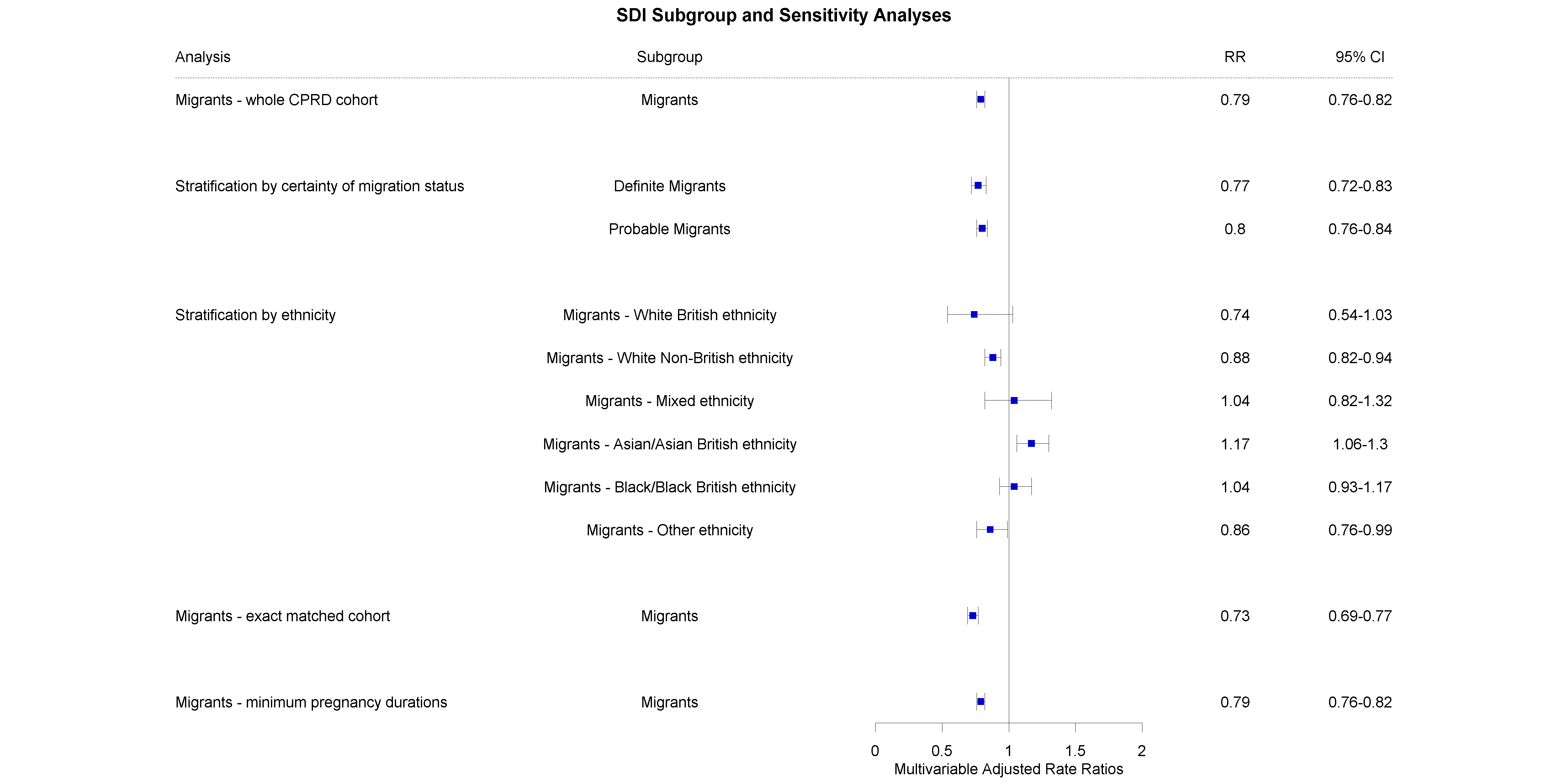


Figure 2.6. Multivariable adjusted rate ratios^†^ across subgroup and sensitivity analyses for POI prescribing in migrant women compared to non-migrant women (CPRD GOLD, 2009-2018)

^†^=adjusted for age group, practice region, deprivation status, and year of study; RR = rate ratio; CI = confidence interval


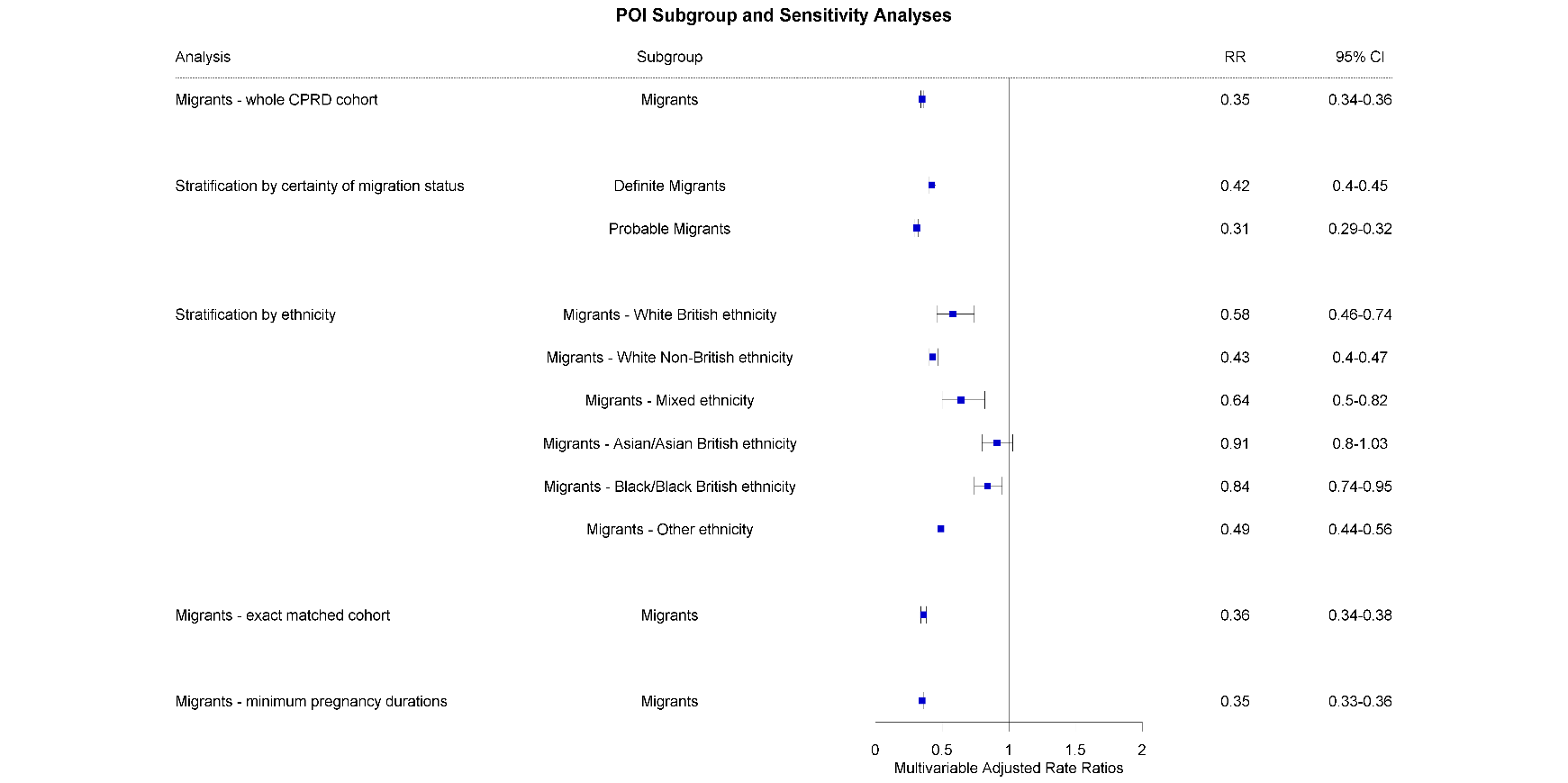

Supplement: Supplementary file 2 [file mmc2.docx]
